# Molecular phylogenetic analysis of *Neritona juttingae* (Mienis, 1973) (Gastropoda, Cycloneritida, Neritidae) with remarks on the phylogenetic position of the genus *Neritona*

**DOI:** 10.3897/zookeys.1269.164112

**Published:** 2026-02-13

**Authors:** Xiuchun Lin, Yuanzheng Meng, Sheng Zeng, Shen Zhong, Yibin Xu, Deyuan Yang

**Affiliations:** 1 College of Environmental and Biological Engineering, Putian University, Putian, China College of Environmental and Biological Engineering, Putian University Putian China; 2 Fujian Provincial Key Laboratory of Ecology-toxicological Effects and Control for Emerging Contaminants, Putian, China College of Ocean and Earth Sciences, Xiamen University Xiamen China; 3 Key Laboratory of Ecological Environment and Information Atlas, Fujian Provincial University, Putian, China Fujian Provincial Key Laboratory of Ecology-toxicological Effects and Control for Emerging Contaminants Putian China; 4 College of Ocean and Earth Sciences, Xiamen University, Xiamen 361102, China Key Laboratory of Ecological Environment and Information Atlas, Fujian Provincial University Putian China; 5 Key Laboratory of Cultivation and High-value Utilization of Marine Organisms in Fujian Province, Fisheries Research Institute of Fujian, Xiamen, Fujian, China Key Laboratory of Cultivation and High-value Utilization of Marine Organisms in Fujian Province, Fisheries Research Institute of Fujian Xiamen China

**Keywords:** 16S rRNA, *COI*, genome skimming, mitogenome, *Neritona juttingae*, phylogenetic analysis, ribosomal RNA gene cluster

## Abstract

In this study, the phylogenetics of an ornamental neritid snail, *Neritona juttingae* (Mienis, 1973), a species native to Southeast Asia and commercially distributed in Chinese markets are studied for the first time. The first complete mitochondrial genome and nuclear ribosomal RNA gene cluster are reported. Phylogenetic trees based on all mitochondrial genes strongly support the division of Neritidae into two subfamilies: Neritinae and Neritininae. Our phylogenetic analysis placed *N. juttingae* within Neritininae, where it forms a distinct evolutionary lineage. The monophyly of the genus *Neritona* remains unresolved because only one species is involved. The relationships among genera within Neritininae were poorly resolved because of the discordant topologies across datasets. Our investigation of mitochondrial gene order in Neritidae revealed highly conserved arrangements. How taxonomic misidentifications and labeling errors in public databases may impact phylogenetic inferences is discussed. This research provides baseline data for aquatic ornamental pet species, contributes to a better understanding of the phylogenetic relationships within Neritoidea and highlights some problems in phylogenetic analysis.

## Introduction

*Neritona juttingae* (Mienis, 1973) is a freshwater gastropod species belonging to the family Neritidae (Cycloneritida: Neritoidea), distributed in Southeast Asia (Mienis 1973; Eichhorst 2016a). It has an atypical appearance, with 6–9 spiral cords on the semi-globose shell, and the cords have a series of small nodules or spines, which makes it very popular among shell collectors and aquarium hobbyists (Eichhorst 2016a, b; Ng et al. 2016). As a result, the species has entered commercial trade and is currently available in ornamental pet markets around the world (Ng et al. 2016; Schäfer 2018; this study).

Despite being popular among hobbyists, hardly any studies on this species exist. The original name of this species is *Nerita aculeata* Gmelin, 1791. Subsequently, Sowerby II (1849) placed it in the genus *Neritina* Lamarck, 1816. Mienis (1973) proposed that the name of this species was preoccupied: *Nerita aculeata* Gmelin, 1791 is a junior homonym of *Nerita aculeata* Muller, 1774 [a junior synonym of *Tympanotonos fuscatus* (Linnaeus, 1758)], and renamed it as *Neritina juttingae* Mienis, 1973 (Eichhorst 2016a). However, *Neritina* was a “dustbin” genus that contained most freshwater or brackish neritid species in the past. Some researchers have proposed different classifications to split *Neritina* into multiple genera or subgenera, but these have not been widely accepted. Eichhorst (2016a, b) proposed a comprehensive classification based on shell morphology and the previous studies after revising all extant Neritidae species. He formally classified Neritidae as two subfamilies with 16 genera: Neritinae Rafinesque, 1815, and Theodoxinae Bandel, 2001 (= Neritininae Poey, 1852). Neritinae contains two marine genera, *Nerita* Linnaeus, 1758, and *Mienerita* Dekker, 2000; Neritininae contains 14 genera, most of which are freshwater or brackish, *Neritona* von Martens, 1869 being one of them.

Currently, the genus *Neritona* comprises only six valid species: *Neritona granosa* (G. B. Sowerby I, 1825); *N. juttingae* (Mienis, 1973); *N. labiosa* (G. B. Sowerby I, 1836) (type species); *N. macgillivrayi* (Reeve, 1855); *N. melanesica* Riech, 1935; *N. plannissima* (Musson, 1869). Von Martens originally established the genus *Neritona* based on the morphology of the operculum of *N. labiosa* (von Martens, 1869). Eichhorst (2016a) redefined the genus, based on the following characters: 1) developed corneous lamella on the operculum; 2) long, thin, and curved apophysis, and long and slender peg; 3) large and semi-globose shell. Most members of this genus are distributed from Southeast Asia to S. Pacific regions, but *N. granosa* is endemic to Hawaii (Eichhorst 2016a).

To date, the monophyletic status and phylogenetic position of this genus remain unexplored based on molecular data. Although several phylogenetic studies based on mitochondrial genomes have been conducted in recent years (Frey and Vermeij 2008; Quintero-Galvis and Raquel-Castro 2013; Sands et al. 2020; Feng et al. 2021; Miao et al. 2022), all of these studies lack sequence data for the genus *Neritona*. Currently, only two studies have provided sequence data for the genus: Alda et al. (2016) sequenced 606 individuals of *N. granosa* to investigate their *COI* haplotypes; Ng et al. (2016) provided the partial mitochondrial cytochrome oxidase subunit I (*COI*) and 16S ribosomal RNA (16S) sequences of *N. juttingae* in an assessment of ornamental freshwater mollusks in Singapore.

In this study, we generated molecular data for *Neritona juttingae* using the genome skimming methods. The molecular data included the complete mitochondrial genome and complete nuclear ribosomal DNA sequences (18S-ITS1-5.8S-ITS2-28S). The phylogenetic position of *Neritona juttingae* was analyzed by combining the new sequence data with all available mitochondrial genomes and partial mitochondrial *COI* and 16S sequences of Neritidae from NCBI, and the monophyly of *Neritona* was also discussed.

## Materials and methods

### Specimen collection, identification, and sequencing

**Figure 1.**
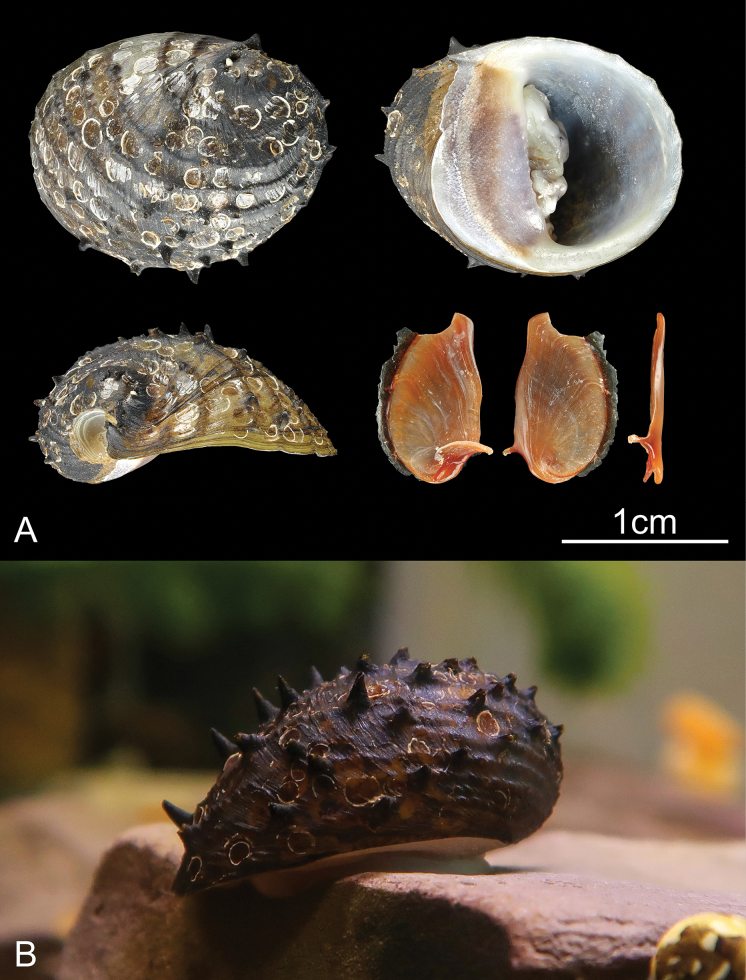
Specimens of *Neritona juttingae*. **A**. RTX-NER-01, shell and operculum; **B**. A living individual of *N. juttingae*. Photographed by Y. Z. M.

The sequenced specimen was bought from Chinese aquarium trading establishement, collected from Sumatra, Indonesia (Fig. 1). Morphological identification followed Eichhorst (2016a). To further confirm the taxonomic identity, we conducted online BLAST analyses (https://blast.ncbi.nlm.nih.gov/Blast.cgi?PROGRAM=blastn&PAGE_TYPE=BlastSearch&LINK_LOC=blasthome) using default parameters, based on the mitochondrial genome assembled from genome skimming data. In addition, we reconstructed phylogenetic trees incorporating all available *COI* and 16S sequences of Neritidae from NCBI. A detailed method of the BLAST analysis is provided in Yang et al. (2024). The specimen was fixed in ethanol (≥ 99.7%), preserved in a -20 °C refrigerator, and finally deposited at the College of the Environment and Ecology, Xiamen University (number: RTX-NER-01).

Specimens were washed with ≥ 97% ethanol before DNA extraction to minimize potential contamination. To avoid intestinal contamination, a tissue sample was clipped from the foot of the specimen. Whole genomic DNA was extracted using the TIANamp Genomic DNA Kit (TIANGEN, Beijing, China). Genomic sequencing was conducted by Novogene Bioinformatics Technology Co., Ltd. (Beijing, China), using a paired-end 150 bp (PE150) strategy on the Illumina NovaSeq X Plus platform. This process yielded 5.60 Gb of raw sequencing data. The raw data were deposited in NCBI under the BioProject accession number PRJNA1394617: BioSample accession SAMN54324965, Sequence Read Archive (SRA) accession SRR36610501.

### Assembly and annotation of mitochondrial genome

The raw paired-end sequencing reads were processed with fastp v. 0.23.4 (Chen et al. 2018) to remove sequencing adapters and trim low-quality regions. To ensure reliable mitochondrial genome assembly, we followed our established assembly pipelines by Yang et al. (2024). In this study, we employed three different assembly tools: (1) NovoPlasty v. 4.3.1 (Dierckxsens et al. 2017), using all mitochondrial genome sequences of Neritidae as seed (NCBI database, September 10, 2024) with default parameters; (2) GetOrganelle v. 1.7.6.1 (Jin et al. 2020), with k-mer thresholds set to 17, 21, 33, 39, 45, 55, 65, 75, 85, 95, 105, 115, and 127, while other parameters were set to default; and (3) FastMitoAssembler (available at https://github.com/suqingdong/FastMitoAssembler). All three methods produced circular mitochondrial genome sequences, differing only in the control region. FastMitoAssembler integrates the approaches of MEANGS, NovoPlasty, and GetOrganelle (Yang et al. 2024), its assembly was ultimately selected, resulting in a mitochondrial genome of 15,950 bp.

The assembled mitochondrial genome was annotated using three tools: MITOS2 (Donath et al. 2019), MitoFinder v. 1.4.1 (Allio et al. 2020), and MitoZ v. 3.6. The generated annotation files (.gb or .gbf) were reorganized with *cox1* (*COI*, *COX1*) as the starting gene using PhyloSuite v.1.2.3 (Zhang et al. 2020). The restructured GenBank (.gb) files were then converted into FASTA format and subjected to tRNA annotation using GeSeq (https://chlorobox.mpimp-golm.mpg.de/geseq.html). Annotations were manually checked using Geneious Prime v. 2022.2.2 (Kearse et al. 2012), following the annotation principles outlined by Yang et al. (2024). The finalized annotation was further confirmed by aligning with mitochondrial genome sequences of closely related species.

**Figure 2.**
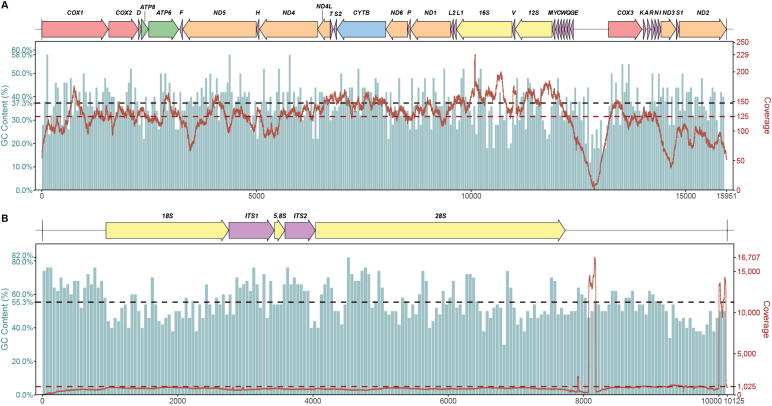
The molecular resources of *Neritona juttingae*. The gene map of mitochondrial genome (**A**) and nuclear rRNA cluster (**B**). The red lines depict the distribution of coverage depth and the green columns depict the GC-content of sequences.

The annotated mitochondrial genome sequence was submitted to the NCBI GenBank database with accession number PP958837. The GC content and nucleotide coverage depth across the mitochondrial genome were calculated using the 'visualize' subcommand in MitoZ v. 3.6 (Meng et al. 2019), and the gene map illustrating the annotated mitochondrial genome was also generated (Fig. 2).

### Assembly of nuclear ribosomal RNA gene cluster

The complete nuclear ribosomal RNA gene cluster (18S-28S rRNA) was assembled using GetOrganelle v. 1.7.6.1 (Jin et al. 2020), with sequence seeding performed using all available 18S and 28S rRNA sequences from Gastropoda downloaded from GenBank as of 15 June 2025. The assembled sequence spans 10,124 bp (accession number: PV833224) with an average coverage depth of 430.9×. Sequence annotation was performed using Geneious Prime v. 9.0.2 with the “Live Annotate & Predict” function enabled. The internal transcribed spacer regions (ITS1 and ITS2) were identified based on the boundaries flanking the 18S, 5.8S, and 28S ribosomal RNA genes.

### Phylogenetic analysis

The newly sequenced mitochondrial genome of *Neritona juttingae* (PP958837) was analyzed alongside all available sequences of Neritidae (36 sequences from the NCBI database, October 1, 2024) using the phylogenetic methods described below, based on a dataset of 13 protein-coding genes (13PCGs). Our analysis revealed that sequences belonging to the same species consistently formed monophyletic groups (Suppl. material 2). We selected a single representative sequence for each species to simplify the analysis. The final dataset, summarized in Table 1, comprised 29 species of Neritidae and two outgroup taxa of Cycloneritida, *Titiscania limacina* (KU342669) and *Georissa bangueyensis* (KU342664), belonging to Neritopsidae and Hydrocenidae (Qi et al. 2023).

**Table 1.** List of 29 species and two outgroups used for phylogenetic analysis.

Species	Family	Length (bp)	A + T (%)	Accession No.	Reference
*Nerita histrio*	Neritidae	15538	65.5	NC_068083	Qi et al. 2023
*Nerita signata*	Neritidae	15630	65.3	NC_068082	Qi et al. 2023
*Nerita plicata*	Neritidae	15737	61.6	NC_068081	Qi et al. 2023
*Nerita ocellata*	Neritidae	15577	63.8	NC_068080	Qi et al. 2023
*Nerita insculpta*	Neritidae	15721	61.5	NC_068079	Qi et al. 2023
*Nerita costata*	Neritidae	15604	61.6	NC_068078	Qi et al. 2023
*Clithon sowerbianum*	Neritidae	15895	64.4	NC_068077	Qi et al. 2023
*Clithon oualaniense*	Neritidae	15712	65.7	NC_068076	Qi et al. 2023
*Clithon retropictum*	Neritidae	15814	64.9	NC_031893	Fukumori et al. 2016
*Nerita japonica*	Neritidae	15875	65.2	MN747116	Feng et al. 2021
*Nerita albicilla*	Neritidae	15314	64.5	MK516738	Feng et al. 2019
*Nerita yoldii*	Neritidae	15719	64.7	MK395169	Xie et al. 2019
*Nerita versicolor*	Neritidae	15866	61.6	KF728890	Arquez et al. 2014
*Nerita tessellata*	Neritidae	15741	64	KF728889	Arquez et al. 2014
*Nerita fulgurans*	Neritidae	15343	64.4	KF728888	Arquez et al. 2014
*Nerita undata*	Neritidae	15878	62.8	NC_087859	Unpublished
*Septaria tessellaria*	Neritidae	15697	65.8	NC_064130	Miao et al. 2022
*Clithon squarrosum*	Neritidae	15905	65	NC_062613	Miao et al. 2022
*Neritina iris*	Neritidae	15618	64.3	NC_062612	Miao et al. 2022
*Clithon corona*	Neritidae	15975	64.8	NC_062611	Miao et al. 2022
*Clithon lentiginosum*	Neritidae	15885	64.8	NC_062610	Miao et al. 2022
*Neripteron violaceum*	Neritidae	15618	65.8	NC_060871	Unpublished
*Theodoxus fluviatilis*	Neritidae	15667	66.1	MT628587	Unpublished
*Nerita balteata*	Neritidae	15571	63.3	MN477253	Unpublished
*Nerita chamaeleon*	Neritidae	15716	65.8	MT161611	Feng et al. 2021
*Neripteron violaceum*	Neritidae	15710	66.3	KY021066	Wang et al. 2019
*Vitta usnea*	Neritidae	15574	64	KU342665	Uribe et al. 2016
*Nerita melanotragus*	Neritidae	15261	63.5	GU810158	Castro and Colgan 2010
*Neritona juttingae*	Neritidae	15950	62.7	PP958837	This study
*Titiscania limacina*	Neritopsidae	15046	61.8	KU342669	Uribe et al. 2016
*Georissa bangueyensis*	Hydrocenidae	15267	68.8	KU342664	Uribe et al. 2016

The extracted protein-coding genes (PCGs) and two rRNAs (2R) from these sequences were aligned using MAFFT in normal mode. Improvement of the multiple sequence alignments for the 13 PCGs was performed using MACSE v. 2 (Ranwez et al. 2018) under the “refinement strategy.” Phylogenetic analyses were conducted on five datasets: (i) 13PCGs123, including all three codon positions of the 13 PCGs; (ii) 13PCGs123+2R, combining the 13PCGs dataset with two rRNA genes; (iii) 13PCGs12, excluding the third codon positions of the 13 PCGs; (iv) 13PCGs12+2R, combining the 13PCGs12 dataset with two rRNA genes; and (v) 13PCGsAA, comprising amino acid sequences translated from the 13 PCGs. To include a broader representation of Neritidae species, sequences of the widely used molecular markers *COI* and 16S rRNA were extracted from GenBank. Data organization was performed using a script developed by Yang et al. (2024). The final combined *COI* and 16S dataset comprised 114 species representing 12 genera of Neritidae, along with two outgroup taxa, totaling 257 *COI* and 257 16S sequences (Suppl. material 1: table SS1). Additionally, more *COI* sequences were used for phylogenetic analysis (*COI*_ex dataset).

TrimAl v. 1.2 (Capella-Gutiérrez et al. 2009) was used to remove ambiguously aligned regions with the ‘automated1’ setting (Suppl. material 1: table SS2). Substitution models were selected using ModelFinder v. 2.2.0 (Kalyaanamoorthy et al. 2017) based on the Bayesian Information Criterion (BIC) for maximum likelihood (ML) analysis and the corrected Akaike Information Criterion (AICc) for Bayesian inference (BI) analysis under a partitioned model (Suppl. material 1: table SS3). ML phylogenetic reconstruction was performed using IQ-TREE v. 2.2.2 (Nguyen et al. 2015; Kalyaanamoorthy et al. 2017) with the best partition scheme and an edge-linked partition model. Node support was evaluated with 200,000 ultrafast bootstraps. BI phylogenies were constructed in MrBayes v. 3.2.7a (Ronquist et al. 2012), with two parallel runs and 2,000,000 generations. Convergence was assessed by the average standard deviation of split frequencies (ASDSF), with additional generations added if the ASDSF value exceeded 0.01.

Topological differences among the inferred phylogenies were evaluated using TreeSpace (Jombart et al. 2017) implemented in R v. 4.3.1 (R Core Team 2023). Finally, the ML and BI trees were visualized using iTOL v. 6 (Letunic and Bork 2024).

### Sequence analyses

Strand asymmetries were calculated using the formulas proposed by Perna and Kocher (1995): AT-skew = (A - T) / (A + T) and GC-skew = (G - C) / (G + C). Codon usage and relative synonymous codon usage (RSCU) of the 13 protein-coding genes (PCGs) were analyzed using PhyloSuite and visualized with the ‘ggplot2’ package (Wickham 2016) in R v. 4.1.3 (R Core Team 2023). DnaSP v. 6.0 (Rozas et al. 2017) was used to estimate the nucleotide diversity (Pi) in a sliding window analysis (a sliding window of 100 bp and a step size of 20 bp) and non-synonymous (Ka) / synonymous (Ks) substitution rates, based on the sequences of Neritidae listed in Table 1.

## Results

### Species identification

The identifying characters of the specimen fit well with *Neritona juttingae*, the description in Eichhorst (2016a), including a semi-globose shell with brown to black periostracum, with 6–9 prominent spiral cords; each spiral cord has a series of small nodules that develop into spines on the final whorl (Fig. 1).

The BLAST result of the complete mitochondrial genome showed no match with *Neritona juttingae* (isolate: ANJ3), previously submitted by Ng et al. (2016). We then extracted the full-length *COI* and 16S sequences from the mitochondrial genome for separate BLAST analyses. The *COI* sequence also did not match with *N. juttingae* (isolate: ANJ3). However, the 16S sequence showed a 99.8% identity with *Neritona juttingae* (isolate: ANJ3; 16S: KU318361.1). Upon further inspection, we found that the *COI* sequence of *N. juttingae* (isolate: ANJ3; *COI*: KU318383.1) is only 313 bp in length. Therefore, we trimmed our *COI* and 16S sequences to a similar length as those in the database before re-running the BLAST analyses. The results then showed high identity (over 99%) for both genes with *N. juttingae* (isolate: ANJ3). All BLAST results see Suppl. material 1: table S4.

Phylogenetic trees based on both *COI* and 16S sequences consistently showed that our specimen clustered with *Neritona juttingae* (isolate: ANJ3). Therefore, based on the combined morphological, BLAST, and phylogenetic evidence, we identified our specimen as *Neritona juttingae*.

### Mitochondrial genome organization

The complete mitochondrial genome of *Neritona juttingae* is 15,950 bp in length, comprising 13 protein-coding genes (PCGs), two rRNA genes, 22 tRNA genes, and a non-coding region (NCR) measuring 818 bp. Six PCGs (*cox1*, *cox2*, *atp8*, *atp6*, *cox3*, and *nad3*) and seven tRNAs (*trnD*, *trnK*, *trnA*, *trnR*, *trnN*, *trnI*, *trnS1*, and *trnT*) are located on the heavy (H-) strand, while the remaining genes are positioned on the light (L-) strand (Table 2, Fig. 2A). Overall, the light- and heavy-strand regions within the mitochondrial genome of *N. juttingae* were concentrated and characterized by both intergenic (19 intergenic intervals, totaling 212 bp) and only one overlapping region (7 bp, between *nad4* and *nad4l*) (Table 2). This typical overlap was common in other Neritidae sequences (Feng et al. 2021; Miao et al. 2022; Qi et al. 2023). All Neritidae mitochondrial genomes exhibited a high A + T content (61.5%–66.3%) (Table 1). The A + T content of *N. juttingae* was 62.7%, with A, T, G, and C constituting 27.8%, 34.9%, 23.0%, and 14.3%, respectively. The AT-skew of the whole mitochondrial genome was negative (-0.113) and the GC-skew was positive (0.235) (Table 3).

**Table 2.** Features of the *N. juttingae* mitochondrial genome.

Gene	Position		Length (bp)	Amino	Start/stop codon	Anticodon	Intergenic region	Strand
	From	To						
*cox1*	1	1548	1548	516	ATG/TAG		11	H
*cox2*	1560	2249	690	230	ATG/TAG		2	H
*trnD*	2252	2317	66			GTC	0	H
*atp8*	2318	2482	165	55	ATG/TAA		6	H
*atp6*	2489	3190	702	234	ATG/TAA		22	H
*trnF*	3213	3280	68			GAA	0	L
*nad5*	3281	4994	1714	572	ATG/T		0	L
*trnH*	4995	5060	66			GTG	9	L
*nad4*	5070	6426	1357	453	ATG/T		-7	L
*nad4l*	6420	6713	294	98	ATG/TAA		4	L
*trnT*	6718	6785	68			TGT	18	H
*trnS2*	6804	6868	65			TGA	5	L
*cytb*	6874	8010	1137	379	ATG/TAA		2	L
*nad6*	8013	8519	507	169	ATG/TAG		1	L
*trnP*	8521	8586	66			TGG	0	L
*nad1*	8587	9519	933	311	ATG/TAA		0	L
*trnL2*	9520	9587	68			TAA	0	L
*trnL1*	9588	9657	70			TAG	0	L
16S	9658	10953	1296				0	L
*trnV*	10954	11021	68			TAC	0	L
12S	11022	11887	866				0	L
*trnM*	11888	11954	67			CAT	14	L
*trnY*	11969	12036	68			GTA	4	L
*trnC*	12041	12104	64			GCA	0	L
*trnW*	12105	12170	66			TCA	0	L
*trnQ*	12171	12239	69			TTG	0	L
*trnG*	12240	12304	65			TCC	12	L
*trnE*	12317	12382	66			TTC	818	L
*cox3*	13201	13980	780	260	ATG/TAA		29	H
*trnK*	14010	14076	67			TTT	33	H
*trnA*	14110	14177	68			TGC	29	H
*trnR*	14207	14275	69			TCG	5	H
*trnN*	14281	14352	72			GTT	2	H
*trnI*	14355	14422	68			GAT	0	H
*nad3*	14423	14776	354	118	ATG/TAG		4	H
*trnS1*	14781	14848	68			GCT	0	H
*nad2*	14849	15950	1102	368	ATG/T		0	H

**Table 3.** Composition and skewness of the *N. juttingae* mitochondrial genome.

Regions	A (%)	T (%)	C (%)	G (%)	A + T (%)	AT-skew	GC-skew	Size (bp)
Full genome	27.8	34.9	14.3	23.0	62.7	-0.113	0.235	15950
PCGs	25.2	36.0	20.0	18.8	61.2	-0.177	-0.032	11280
tRNAs	31.1	32.3	15.3	21.3	63.4	-0.018	0.164	1482
rRNAs	36.2	29.4	17.3	17.1	65.6	0.103	-0.005	2162
1^st^ codon	25.9	29.7	18.1	26.4	55.6	-0.068	0.185	3760
2^nd^ codon	18.1	42.8	22.7	16.4	60.9	-0.407	-0.162	3760
3^rd^ codon	31.6	35.5	19.2	13.6	67.1	-0.058	-0.172	3760
12S	35.2	28.9	17.0	18.9	64.1	0.099	0.055	866
16S	36.8	29.8	17.5	15.9	66.6	0.105	-0.048	1296
*atp6*	20.1	42.2	15.8	21.9	62.3	-0.355	0.162	702
*atp8*	25.5	41.2	12.1	21.2	66.7	-0.236	0.273	165
*cox1*	20.6	40.2	15.9	23.3	60.8	-0.323	0.188	1548
*cox2*	23.3	37.2	14.6	24.8	60.5	-0.230	0.257	690
*cox3*	21.0	38.7	14.9	25.4	59.7	-0.296	0.261	780
*cytb*	28.1	31.2	25.8	15.0	59.3	-0.053	-0.266	1137
*nad1*	28.8	33.0	23.4	14.8	61.8	-0.068	-0.225	933
*nad2*	19.2	42.6	10.7	27.4	61.8	-0.378	0.438	1102
*nad3*	15.5	46.6	9.3	28.5	62.1	-0.500	0.507	354
*nad4*	29.8	32.3	26.4	11.6	62.1	-0.040	-0.390	1357
*nad4l*	33.0	28.9	22.1	16.0	61.9	0.066	-0.161	294
*nad5*	29.7	30.2	27.4	12.7	59.9	-0.009	-0.368	1714
*nad6*	29.6	35.3	21.7	13.4	64.9	-0.088	-0.236	507

### Nuclear rRNA gene cluster

The nuclear rRNA gene cluster (10,124 bp) contains the full complement of ribosomal RNA genes: 18S rRNA (1,818 bp), ITS1 (673 bp), 5.8S rRNA (156 bp), ITS2 (453 bp), and 28S rRNA (3,693 bp) (Fig. 2B).

### Genes and codon usage

The 13 protein-coding genes (PCGs) of *Neritona juttingae* have a total length of 11,280 bp and encode 3,760 amino acids. All 13 PCGs begin with the ATG start codon. Regarding stop codons, *nad5*, *nad4*, and *nad2* terminate with a single T, *cox1*, *cox2*, *nad6*, and *nad3* end with TAG, while the remaining genes end with TAA (Table 2). The AT- and GC-skews of the 13 PCGs are negative, at -0.177 and -0.032, respectively (Table 3). Six PCGs (*cytb*, *nad1*, *nad4*, *nad4l*, *nad5*, and *nad6*) exhibit negative GC-skew values, whereas the remaining seven PCGs show positive values. The 12S rRNA gene (866 bp) is located between the *trnV* and *trnM* genes, while the 16S rRNA gene (1,296 bp) is positioned between *trnL1* and *trnV* (Table 2, Fig. 2A). A total of 22 tRNA genes, ranging in length from 64 to 72 bp, were identified in the mitochondrial genome of *N. juttingae*.

**Figure 3.**
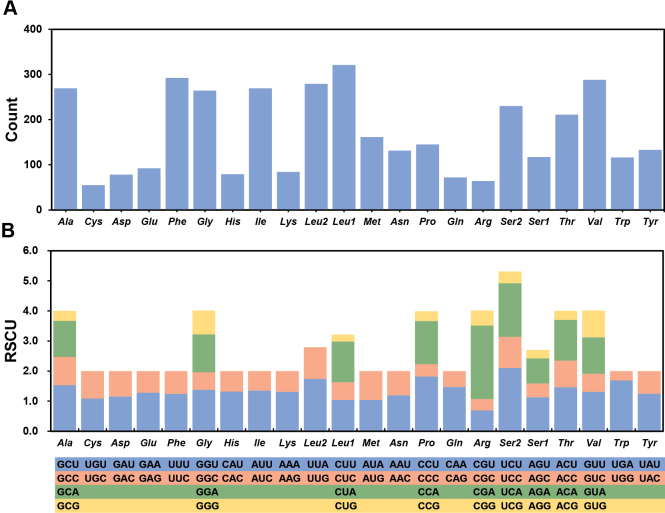
Amino acid composition (**A**) and relative synonymous codon usage (**B**) of the *N. juttingae* mitochondrial genome. The codon families are provided under the x-axis.

The relative synonymous codon usage (RSCU) values for the PCGs in the mitochondrial genome were also analyzed (Fig. 3, Suppl. material 1: table S5). The most frequently used amino acids are *Leu* (16.0%), *Ser* (9.25%), *Phe* (7.79%), and *Val* (7.68%). The least common amino acids are *Cys* (1.47%), *Arg* (1.71%), *Gln* (1.92%), and *Asp* (2.08%) (Fig. 3A, Suppl. material 1: table S5). RSCU analysis indicates that the most frequently used codons are CGA (*Arg*), UCU (*Ser*), and CCU (*Pro*), while CUG (*Leu*), AGG (*Ser*), and ACG (*Thr*) have the lowest frequencies (Fig. 3B, Suppl. material 1: table S5).

### Phylogenetic analysis

**Figure 4.**
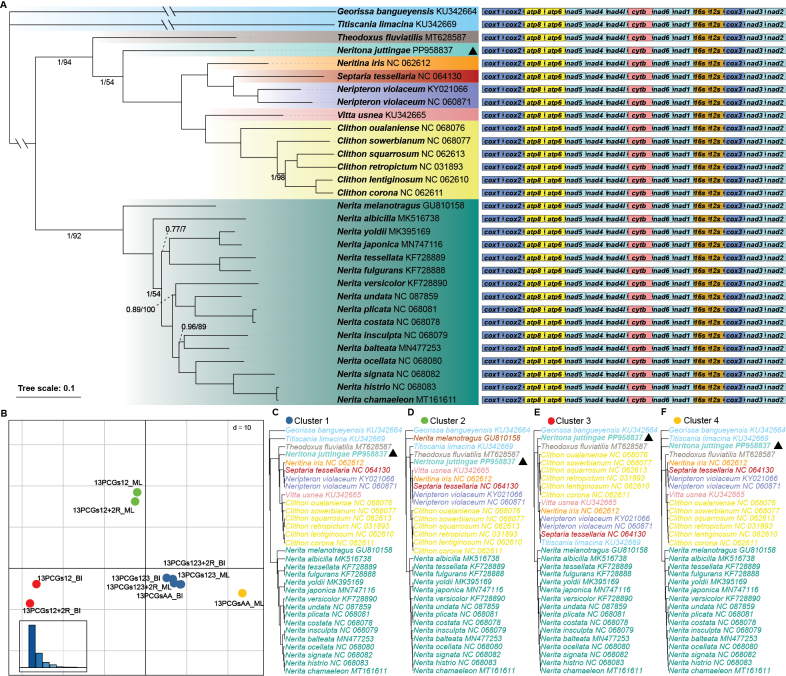
An analysis of phylogenies from five mitochondrial genome datasets. **A**. Maximum likelihood (ML) and Bayesian inference (BI) tree of Neritidae based on the dataset 13PCGs123. The GenBank accession numbers used are listed after the species names. The scale bar (0.1) corresponds to the estimated number of substitutions per site. Numbers at nodes are statistical support values for “BI posterior probabilities/ML bootstrap support”. “Unlabeled” denotes 100% bootstrap support. Color-coded clades are different genera within Neritidae. The gene order is shown to the right (13 PCGs and two rRNAs); **B**. Two-dimensional MDS plot of ten trees, colored by different clusters; **C–F**. For each cluster identified in (**B**), a representative tree was selected.

The gene arrangement of the 13 PCGs and 2 rRNAs within the family Neritidae is completely consistent (Fig. 4A, Suppl. material 3) and cannot provide useful phylogenetic information. A total of ten ML and BI trees were inferred from five mitochondrial genome datasets of Neritidae. These ten trees were grouped into four clusters, representing four distinct tree types (see Fig. 4B–F, Suppl. material 4). The most common topology, Cluster 1, is shown in Fig. 4A, C. The differences between Cluster 1 and Clusters 2–3 mainly lie in the sequences of *Vitta usnea* (KU342665), *Nerita melanotragus* (GU810158), and the outgroup species *Titiscania limacina* (KU342669). The relationships within genera are generally consistent except for *Nerita melanotragus* (GU810158), which forms a distinct clade in Cluster 2. Three genera, *Neritina*, *Septaria*, and *Neripteron*, clustered together with each other in all four clusters. The positions of *Theodoxus fluviatilis* (MT628587) and *Neritona juttingae* (PP958837) form two distinct lineages from other genera, although there are some differences between the four clusters (Fig. 4).

Subsequent phylogenetic interpretations were based on the dataset 13PCGs123. Phylogenetic analyses using the 13PCGs123 dataset from the available mitochondrial genomes (29 taxa in Neritidae, including 8 genera) support that *Neritona juttingae* belongs to a separate genus.

The phylogenetic tree comprises 29 sequences from 28 species of Neritidae, belonging to eight genera (Fig. 4A). The tree can be divided into two primary clades: Clade A comprises seven genera (*Theodoxus*, *Neritona*, *Neritina*, *Septaria*, *Neripteron*, *Vitta*, and *Clithon*), and Clade B comprises only one genus, *Nerita*. The genus *Neritona* is located inside Clade A, forming a distinct clade, which is most closely related to a clade comprising five freshwater or brackish genera (*Neritina*, *Septaria*, *Neripteron*, *Vitta*, and *Clithon*) with strong support (BS = 100%, PP = 1).

**Figure 5.**
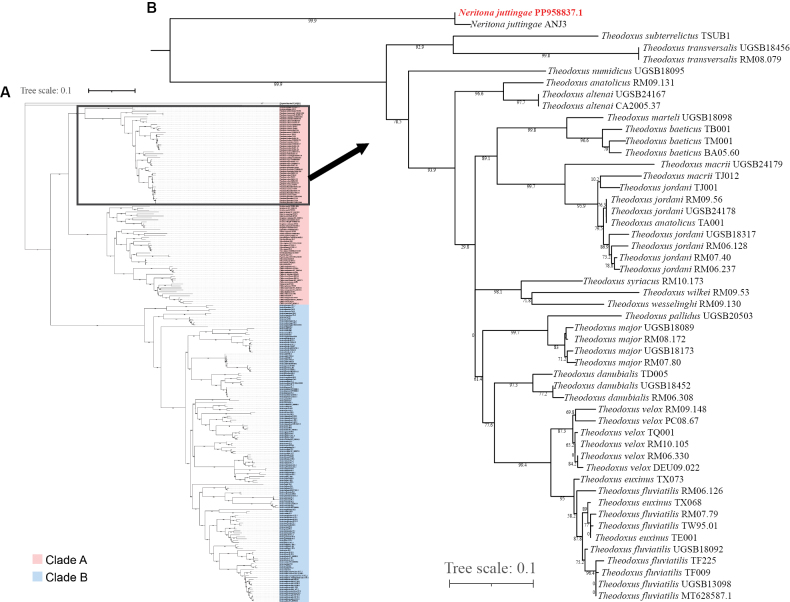
**A**. ML tree based on the *COI+*16S dataset (Suppl. material 5); **B**. Enlarged version of the branch containing *Neritona juttingae* sequences. The *N. juttingae* sequence reported in this study are highlighted in red text.

The *COI+*16S tree includes 114 species from 12 genera of Neritidae, and two outgroups (Fig. 5, Suppl. material 5). The structure of the *COI+*16S tree is similar to the mitochondrial genome tree, and both can be divided into two clades, comprising about the same genera. Two sequences of *Neritona juttingae* are also located inside Clade A, forming a distinct clade, which is most closely related to the other 10 genera of Clade A.

The *COI*_ex tree includes 1480 sequences of Neritidae. This tree is not clustered into two distinct branches like the previous two, but the genus *Nerita* still forms a generally distinct clade. Two sequences of *N. juttingae* form a distinct clade, too. Sequences of the genus *Neritona* do not cluster together. Two sequences of *N. juttingae* form a distinct group close to the genus *Theodoxus*, one sequence of *N. granosa* (AF364065) is close to *Neripteron vespertinum*AF364066, and other *N. granosa* sequences form a monophyletic group (Suppl. material 6).

### Nucleotide diversity and evolutionary rate analysis

**Figure 6.**
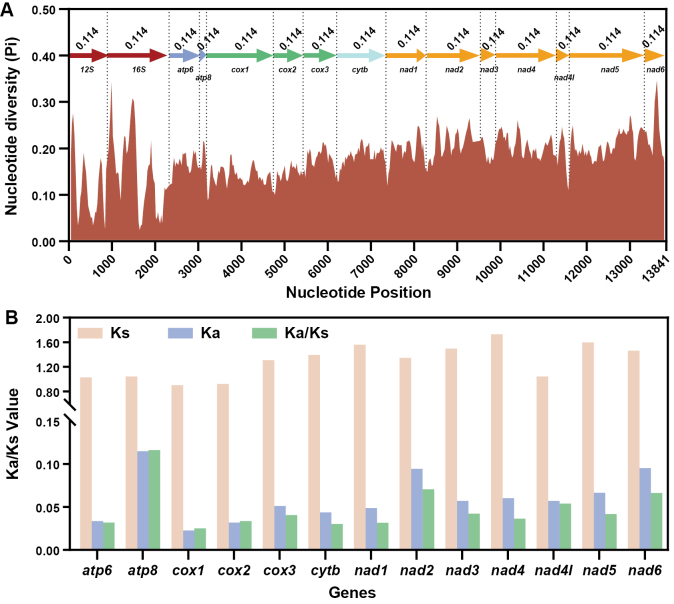
Nucleotide diversity analysis (**A**) of two rRNAs + 13 PCGs and Ka/Ks rates (**B**) of 13 PCGs based on 29 Neritidae species. The Pi values for the 13 PCGs + two rRNAs are shown on the graph. The red line represents the value of nucleotide diversity (Pi) (window size = 100 bp, step size = 20 bp). The pink, purple, and green columns represent the values of Ks, Ka, and Ka/Ks, respectively.

The nucleotide diversity (Pi) analysis was conducted using concatenated alignments of 13 PCGs and two rRNAs of 29 Neritidae species. The sequence variation ratio exhibits variable nucleotide diversity between the Neritidae, with Pi values for the 100 bp windows ranging from 0.024 to 0.346. 12S (Pi = 0.114), *cox1* (0.142), 16S (0.145), and *cox2* (0.148) exhibit a comparatively low sequence variability, whereas *nad6* (0.231), *nad2* (0.214), *nad4* (0.208), and *nad5* (0.208) have a comparatively high sequence variability (Fig. 6A). This is broadly corroborated by the non-synonymous/synonymous (Ka/Ks) ratio analysis, which shows that *cox1* (Ka/Ks = 0.025), *cytb* (0.030), *nad1* (0.032), and *atp6* (0.032) are evolving comparatively slowly, whereas *atp8* (0.116), *nad2* (0.070), and *nad6* (0.066) are evolving comparatively fast (Fig. 6B).

## Discussion

In this study, our results well supported the classification of this family into two subfamilies (Neritininae and Neritinae), which is consistent with previous mitochondrial genome-based phylogenetic analysis studies (Feng et al. 2021; Miao et al. 2022; Qi et al. 2023). These studies consistently reveal two distinct clades: one clade (Fig. 4, Clade A) includes genera of the subfamily Neritininae, which primarily inhabit freshwater or brackish environments and typically exhibit amphidromous life histories; the other clade (Fig. 4, Clade B) consists exclusively of the marine genus *Nerita*, representing the subfamily Neritinae (Ford 1979; Eichhorst 2016a, b).

However, within Neritininae, the phylogenetic relationships remain unstable, as inconsistencies are observed across our five mitochondrial genome datasets (Fig. 4). These discrepancies may be attributable to heterogeneous evolutionary rates among mitochondrial genes (Mueller 2006), long-branch attraction (Masta et al. 2010), and incomplete lineage sorting (Ballesteros and Sharma 2019). Increasing the number of species in the sample is expected to alleviate the long branch attraction phenomenon (Ontano et al. 2021) and help detect incomplete phylogenetic sorting (Maddison and Knowles 2006). Four clusters of tree structure were concluded from the ten trees based on five datasets. The relationship between genera varies a lot, although the genus-level clades are generally stable among the four clusters. The relatively stable relationship between *Neritina*, *Septaria*, and *Neripteron* suggested the three genera might have more closer relationship with each other than with other genera. Notably, the distinct position of *Nerita melanotragus* (GU810158) in Cluster 2 is similar to Feng et al. (2021). The mitochondrial gene order is highly conserved within Neritidae, which cannot provide phylogenetic information. These results may suggest the limitation of mitochondrial genome in explaining the relationship within Neritidae. Moreover, the current phylogenetic trees are still insufficient to explain the relationship between habitat and evolution in Neritidae. Holthuis (1995) proposed that there are at least 12 shifts between marine, brackish, and freshwater habitats in Cycloneritida. Qi et al. (2023) also mentioned that Neritidae moved repeatedly between different habitats. To solve this problem, importing more taxa and integrating nuclear genomic data is still required to explain the phylogenetics of all sixteen genera of Neritidae.

The monophyly of the genus *Neritona* could not be robustly assessed in this study due to the inclusion of only a single species (*N. juttingae*) within the molecular dataset. Phylogenetic analyses consistently placed *Neritona juttingae* as a distinct lineage within Clade A in both mitochondrial genome and *COI+*16S trees (Figs 4, 5), suggesting *N. juttingae* belongs to a separate genus of Neritininae. Considering that *N. juttingae* is the only species of this genus in the tree, as well as the unusually wide distribution of species in the genus (from Southeast Asia to Hawaii) and the large morphological differences within the genus, monophyly of the genus *Neritona* could not be evaluated due to the inclusion of only one of its six valid species (*N. juttingae*) in our analysis. The distinct phylogenetic position of *N. juttingae* suggests that the genus, as currently defined, may not be monophyletic, but this hypothesis requires testing with data from the type species (*N. labiosa*) and other congeners. Eichhorst (2016a) even hypothesized that *N. juttingae* might belong to a new genus distinct from *Neritona* due to its unique appearance, because it is the only nerite species with multiple rows of spines. This assumption is possible because the sequences of *N. juttingae* and *N. granosa* do not cluster together in the *COI*_ex tree. However, although the *COI*_ex dataset contains many sequences, it provides limited phylogenetic resolution due to their short lengths. In summary, additional sampling and more complete mitochondrial genomes are needed in future studies to clarify the phylogenetic relationships of *N. juttingae*, and potentially, the entire genus *Neritona*.

We noticed that the two sequences of *Neripteron violaceum* (Gmelin, 1791) (KY021066 and NC_060871) exhibit an unusual level of consistency (89.94% identity), and the two sequences in the *COI+*16S tree are located in different subclades. KY021066 is clustered with the other two *N. violaceum* sequences, and NC_060871 is clustered with an *N. auriculatum* sequence. Therefore, we suppose the two sequences may not correspond to the same species. This phenomenon suggests there is a misidentification or cryptic species. Since the references for both sequences do not provide specimen images and morphological descriptions, this problem cannot be resolved at present. Given that some sequences in the database have been proven to be misidentified (Xu et al. 2024; Xu et al. 2025), we recommend that when uploading sequences to the database, the references should be accompanied by clear images, morphological descriptions, and acquisition information of the specimens. Besides, three of the sequences were incorrectly labeled as their invalid synonyms in the GenBank: *Neripteron violaceum* (KY021066) is labeled as *Neritina violacea*, belonging to another genus (Eichhorst 2016b), and *Septaria tessellaria* (NC064130) and *Nerita signata* (NC068082) are labeled as *S. lineata* and *N. reticulata*, respectively (MolluscaBase 2025). Similar situations are also observed in the *COI*_ex dataset. Incorrectly labeled and misidentified sequences in databases may lead to erroneous phylogenetic results. Here, we recommend that the sequences in the public databases be checked carefully before phylogenetic analysis, especially their identification and taxonomic status. Our study provided definitive molecular references for *N. juttingae*, which is particularly relevant for the ornamental pet trade, where accurate species identification is fundamental to sustainable sourcing, consumer transparency, and effective conservation management of popular species.

## Additional information

### Use of AI

No use of AI was reported.

### Author ORCIDs

Yuanzheng Meng https://orcid.org/0009-0006-3294-8973

Sheng Zeng https://orcid.org/0009-0001-0943-3772

Shen Zhong https://orcid.org/0009-0008-4353-5052

Yibin Xu https://orcid.org/0009-0004-7386-806X

Deyuan Yang https://orcid.org/0000-0003-3735-9909

### Data availability

All of the data that support the findings of this study are available in the main text or Supplementary Information.

## Supplementary materials

Supplementary material 1 Supplementary tables Data type xlsx Explanation note **table SS1**. List of 257 sequences used for *COI+*16S dataset; **table SS2**. Original and TrimAl lengths of 13 PCGs, 2 rRNAs, and 13 PCGsAA sequences; **table SS3**. Best partitioning schemes and models based on different datasets for maximum likelihood and Bayesian inference analysis; **table S4**. BLAST results of different genes or region (only show top 10 results); **table S5**. Codon numbers and relative synonymous codon usage (RSCU) of 13 PCGs in the *Neritona juttingae* mitochondrial genome. https://binary.pensoft.net/file/1538508This dataset is made available under the Open Database License (http://opendatacommons.org/licenses/odbl/1.0/). The Open Database License (ODbL) is a license agreement intended to allow users to freely share, modify, and use this Dataset while maintaining this same freedom for others, provided that the original source and author(s) are credited. Xiuchun Lin, Yuanzheng Meng, Sheng Zeng, Shen Zhong, Yibin Xu, Deyuan Yang

Supplementary material 2 ML and BI from all NCBI mitochondrial genomes (2025-01-10) of Neritidae Data type pdf https://binary.pensoft.net/file/1538509This dataset is made available under the Open Database License (http://opendatacommons.org/licenses/odbl/1.0/). The Open Database License (ODbL) is a license agreement intended to allow users to freely share, modify, and use this Dataset while maintaining this same freedom for others, provided that the original source and author(s) are credited. Xiuchun Lin, Yuanzheng Meng, Sheng Zeng, Shen Zhong, Yibin Xu, Deyuan Yang

Supplementary material 3 All the mt genes order of Neritidae Data type pdf https://binary.pensoft.net/file/1538510This dataset is made available under the Open Database License (http://opendatacommons.org/licenses/odbl/1.0/). The Open Database License (ODbL) is a license agreement intended to allow users to freely share, modify, and use this Dataset while maintaining this same freedom for others, provided that the original source and author(s) are credited. Xiuchun Lin, Yuanzheng Meng, Sheng Zeng, Shen Zhong, Yibin Xu, Deyuan Yang

Supplementary material 4 10 trees from different datasets and tree-building methods Data type zip https://binary.pensoft.net/file/1538511This dataset is made available under the Open Database License (http://opendatacommons.org/licenses/odbl/1.0/). The Open Database License (ODbL) is a license agreement intended to allow users to freely share, modify, and use this Dataset while maintaining this same freedom for others, provided that the original source and author(s) are credited. Xiuchun Lin, Yuanzheng Meng, Sheng Zeng, Shen Zhong, Yibin Xu, Deyuan Yang

Supplementary material 5 ML tree based on the *COI*+16S dataset Data type pdf https://binary.pensoft.net/file/1538512This dataset is made available under the Open Database License (http://opendatacommons.org/licenses/odbl/1.0/). The Open Database License (ODbL) is a license agreement intended to allow users to freely share, modify, and use this Dataset while maintaining this same freedom for others, provided that the original source and author(s) are credited. Xiuchun Lin, Yuanzheng Meng, Sheng Zeng, Shen Zhong, Yibin Xu, Deyuan Yang

Supplementary material 6 ML tree based on the *COI_ex* dataset Data type pdf https://binary.pensoft.net/file/1538513This dataset is made available under the Open Database License (http://opendatacommons.org/licenses/odbl/1.0/). The Open Database License (ODbL) is a license agreement intended to allow users to freely share, modify, and use this Dataset while maintaining this same freedom for others, provided that the original source and author(s) are credited. Xiuchun Lin, Yuanzheng Meng, Sheng Zeng, Shen Zhong, Yibin Xu, Deyuan Yang

## References

[B1] Alda F, Gagne RB, Walter RP, Hogan JD, Moody KN, Zink F, McIntyre PB, Gilliam JF, Blum MJ (2016) Colonization and demographic expansion of freshwater fauna across the Hawaiian archipelago. Journal of Evolutionary Biology 29(10): 2054–2069. 10.1111/jeb.12929 27369460

[B2] Allio R, Schomaker‐Bastos A, Romiguier J, Prosdocimi F, Nabholz B, Delsuc F (2020) MitoFinder: Efficient automated large‐scale extraction of mitogenomic data in target enrichment phylogenomics. Molecular Ecology Resources 20(4): 892–905. 10.1111/1755-0998.13160 PMC7497042 32243090

[B3] Arquez M, Colgan D, Castro LR (2014) Sequence and comparison of mitochondrial genomes in the genus *Nerita* (Gastropoda: Neritimorpha: Neritidae) and phylogenetic considerations among gastropods. Marine Genomics 15: 45–54. 10.1016/j.margen.2014.04.007 24798873

[B4] Ballesteros JA, Sharma PP (2019) A critical appraisal of the placement of *Xiphosura* (Chelicerata) with account of known sources of phylogenetic error. Systematic Biology 68(6): 896–917. 10.1093/sysbio/syz011 30917194

[B5] Capella-Gutiérrez S, Silla-Martínez JM, Gabaldón T (2009) trimAl: A tool for automated alignment trimming in large-scale phylogenetic analyses. Bioinformatics (Oxford, England) 25(15): 1972–1973. 10.1093/bioinformatics/btp348 PMC2712344 19505945

[B6] Castro LR, Colgan D (2010) The phylogenetic position of Neritimorpha based on the mitochondrial genome of *Nerita melanotragus* (Mollusca: Gastropoda). Molecular Phylogenetics and Evolution 57(2): 918–923. 10.1016/j.ympev.2010.08.030 20817109

[B7] Chen S, Zhou Y, Chen Y, Gu J (2018) fastp: An ultra-fast all-in-one FASTQ preprocessor. Bioinformatics (Oxford, England) 34(17): i884–i890. 10.1093/bioinformatics/bty560 PMC6129281 30423086

[B8] Dierckxsens N, Mardulyn P, Smits G (2017) NOVOPlasty: de novo assembly of organelle genomes from whole genome data. Nucleic Acids Research 45(4): e18–e18. 10.1093/nar/gkw955 PMC5389512 28204566

[B9] Donath A, Jühling F, Al-Arab M, Bernhart SH, Reinhardt F, Stadler PF, Middendorf M, Bernt M (2019) Improved annotation of protein-coding genes boundaries in metazoan mitochondrial genomes. Nucleic Acids Research 47(20): 10543–10552. 10.1093/nar/gkz833 PMC6847864 31584075

[B10] Eichhorst TE (2016a) Neritidae of the World. Vol. 2, Conchbooks, Harxheim, 672 pp.

[B11] Eichhorst TE (2016b) Neritidae of the World. Vol. 1, Conchbooks, Harxheim, 694 pp.

[B12] Feng J, Fu Z, Guo Y, Ye Y, Li J, Guo B, Lü Z (2019) The complete mitochondrial genome of *Nerita albicilla* (Neritimorpha: Neritidae). Mitochondrial DNA,. Part B, Resources 4(1): 1597–1598. 10.1080/23802359.2019.1601523

[B13] Feng J, Xia L, Yan C, Miao J, Ye Y-y, Li J, Guo B-y, Lü Z (2021) Characterization of four mitochondrial genomes of family Neritidae (Gastropoda: Neritimorpha) and insight into its phylogenetic relationships. Scientific Reports 11(1): 11748. 10.1038/s41598-021-91313-0 PMC8175686 34083683

[B14] Ford JI (1979) Biology of a Hawaiian Fluvial Gastropod: *Neritina granosa* Sowerby (Prosobranchia: Neritidae). Master’s thesis, Hawaii: University of Hawai’i at Manoa, 94 pp.

[B15] Frey MA, Vermeij GJ (2008) Molecular phylogenies and historical biogeography of a circumtropical group of gastropods (Genus: *Nerita*): implications for regional diversity patterns in the marine tropics. Molecular Phylogenetics and Evolution 48(3): 1067–1086. 10.1016/j.ympev.2008.05.009 18586528

[B16] Fukumori H, Itoh H, Kano Y (2016) The complete mitochondrial genome of the stream snail *Clithon retropictum* (Neritimorpha: Neritidae). Mitochondrial DNA, Part B, Resources 1(1): 820–821. 10.1080/23802359.2016.1247659 PMC7799950 33473640

[B17] Holthuis BV (1995) Evolution between marine and freshwater habitats: a case study of the gastropod suborder Neritopsina. Doctoral dissertation, Washington: University of Washington, 286 pp.

[B18] Jin J-J, Yu W-B, Yang J-B, Song Y, DePamphilis CW, Yi T-S, Li D-Z (2020) GetOrganelle: A fast and versatile toolkit for accurate de novo assembly of organelle genomes. Genome Biology 21(1): 1–31. 10.1186/s13059-020-02154-5 PMC7488116 32912315

[B19] Jombart T, Kendall M, Almagro‐Garcia J, Colijn C (2017) treespace: Statistical exploration of landscapes of phylogenetic trees. Molecular Ecology Resources 17(6): 1385–1392. 10.1111/1755-0998.12676 PMC5724650 28374552

[B20] Kalyaanamoorthy S, Minh BQ, Wong TK, Von Haeseler A, Jermiin LS (2017) ModelFinder: Fast model selection for accurate phylogenetic estimates. Nature Methods 14(6): 587–589. 10.1038/nmeth.4285 PMC5453245 28481363

[B21] Kearse M, Moir R, Wilson A, Stones-Havas S, Cheung M, Sturrock S, Buxton S, Cooper A, Markowitz S, Duran C, Thierer T, Ashton B, Meintjes P, Drummond A (2012) Geneious Basic: An integrated and extendable desktop software platform for the organization and analysis of sequence data. Bioinformatics (Oxford, England) 28(12): 1647–1649. 10.1093/bioinformatics/bts199 PMC3371832 22543367

[B22] Letunic I, Bork P (2024) Interactive Tree of Life (iTOL) v6: Recent updates to the phylogenetic tree display and annotation tool. Nucleic Acids Research 52(W1): W78–W82. 10.1093/nar/gkae268 PMC11223838 38613393

[B23] Maddison WP, Knowles LL (2006) Inferring phylogeny despite incomplete lineage sorting. Systematic Biology 55(1): 21–30. 10.1080/10635150500354928 16507521

[B24] Masta SE, McCall A, Longhorn SJ (2010) Rare genomic changes and mitochondrial sequences provide independent support for congruent relationships among the sea spiders (Arthropoda, Pycnogonida). Molecular Phylogenetics and Evolution 57(1): 59–70. 10.1016/j.ympev.2010.06.020 20601005

[B25] Meng G, Li Y, Yang C, Liu S (2019) MitoZ: A toolkit for animal mitochondrial genome assembly, annotation and visualization. Nucleic Acids Research 47(11): e63. 10.1093/nar/gkz173 PMC6582343 30864657

[B26] Miao J, Feng J, Liu X, Yan C, Ye Y, Li J, Xu K, Guo B, Lü Z (2022) Sequence comparison of the mitochondrial genomes of five brackish water species of the family Neritidae: Phylogenetic implications and divergence time estimation. Ecology and Evolution 12(6): e8984. 10.1002/ece3.8984 PMC9170520 35784089

[B27] Mienis HK (1973) Notes on recent and fossil Neritidae. 3. *Neritina juttingae*, new name for *Nerita aculeata* Gmelin, 1791, non Müller, 1774 (Mollusca, Gastropoda). Basteria 37: 39–40. http://natuurtijdschriften.nl/download?type=document;docid=596591

[B28] MolluscaBase (2025) MolluscaBase eds. https://www.molluscabase.org [accessed 3 March 2025]

[B29] Mueller RL (2006) Evolutionary rates, divergence dates, and the performance of mitochondrial genes in Bayesian phylogenetic analysis. Systematic Biology 55(2): 289–300. 10.1080/10635150500541672 16611600

[B30] Ng TH, Tan SK, Wong WH, Meier R, Chan S-Y, Tan HH, Yeo DC (2016) Molluscs for sale: Assessment of freshwater gastropods and bivalves in the ornamental pet trade. PLOS ONE 11(8): e0161130. 10.1371/journal.pone.0161130 PMC4985174 27525660

[B31] Nguyen L-T, Schmidt HA, Von Haeseler A, Minh BQ (2015) IQ-TREE: A fast and effective stochastic algorithm for estimating maximum-likelihood phylogenies. Molecular Biology and Evolution 32(1): 268–274. 10.1093/molbev/msu300 PMC4271533 25371430

[B32] Ontano AZ, Gainett G, Aharon S, Ballesteros JA, Benavides LR, Corbett KF, Gavish-Regev E, Harvey MS, Monsma S, Santibáñez-López CE, Setton EVW, Zehms JT, Zeh JA, Zeh DW, Sharma PP (2021) Taxonomic sampling and rare genomic changes overcome long-branch attraction in the phylogenetic placement of pseudoscorpions. Molecular Biology and Evolution 38(6): 2446–2467. 10.1093/molbev/msab038 PMC8136511 33565584

[B33] Perna NT, Kocher TD (1995) Patterns of nucleotide composition at fourfold degenerate sites of animal mitochondrial genomes. Journal of Molecular Evolution 41(3): 353–358. 10.1007/BF01215182 7563121

[B34] Qi L, Xu B, Kong L, Li Q (2023) Improved phylogenetic resolution within Neritidae (Gastropoda, Neritimorpha) with implications for the evolution of shell traits and habitat. Zoologica Scripta 52(1): 46–57. 10.1111/zsc.12567

[B35] Quintero-Galvis J, Raquel-Castro L (2013) Molecular phylogeny of the Neritidae (Gastropoda: Neritimorpha) based on the mitochondrial genes cytochrome oxidase I (COI) and 16S rRNA. Acta Biologica Colombiana 18(2): 307–318. http://www.redalyc.org/articulo.oa?id=319028011007

[B36] R Core Team (2023) R: A language and environment for statistical computing. https://www.R-project.org/ [accessed 15 May 2025]

[B37] Ranwez V, Douzery EJ, Cambon C, Chantret N, Delsuc F (2018) MACSE v2: Toolkit for the alignment of coding sequences accounting for frameshifts and stop codons. Molecular Biology and Evolution 35(10): 2582–2584. 10.1093/molbev/msy159 PMC6188553 30165589

[B38] Ronquist F, Teslenko M, Van Der Mark P, Ayres DL, Darling A, Höhna S, Larget B, Liu L, Suchard MA, Huelsenbeck JP (2012) MrBayes 3.2: Efficient Bayesian phylogenetic inference and model choice across a large model space. Systematic Biology 61(3): 539–542. 10.1093/sysbio/sys029 PMC3329765 22357727

[B39] Rozas J, Ferrer-Mata A, Sánchez-DelBarrio JC, Guirao-Rico S, Librado P, Ramos-Onsins SE, Sánchez-Gracia A (2017) DnaSP 6: DNA sequence polymorphism analysis of large data sets. Molecular Biology and Evolution 34(12): 3299–3302. 10.1093/molbev/msx248 29029172

[B40] Sands AF, Glöer P, Gürlek ME, Albrecht C, Neubauer TA (2020) A revision of the extant species of *Theodoxus* (Gastropoda, Neritidae) in Asia, with the description of three new species. Zoosystematics and Evolution 96(1): 25–66. 10.3897/zse.96.48312

[B41] Schäfer F (2018) *Neritina juttingae*. https://www.aquariumglaser.de/en/31-crayfishes-shrimps-crabs-snails-mussels/neritina-juttingae-2/ [accessed June 23.2025]

[B42] Sowerby GB II (1849) Monograph of the genus *Neritina*. In: Sowerby GBI (Ed.) Thesaurus conchyliorum, or monographs of genera of shells. Privately published, London, 507–546 [pls 109-116]. https://biodiversitylibrary.org/page/11075613

[B43] Uribe JE, Colgan D, Castro LR, Kano Y, Zardoya R (2016) Phylogenetic relationships among superfamilies of Neritimorpha (Mollusca: Gastropoda). Molecular Phylogenetics and Evolution 104: 21–31. 10.1016/j.ympev.2016.07.021 27456746

[B44] von Martens E (1869) Über die Deckel der Schneckengattungen *Neritina*, *Nerita*, und *Navicella*. Sitzungsberichte der Gesellschaft naturforschender Freude zu Berlin: 21–23. https://www.biodiversitylibrary.org/page/7915090

[B45] Wang P, Zhu P, Wu H, Xu Y, Liao Y, Zhang H (2019) The complete mitochondrial genome of *Neritina violacea*. Mitochondrial DNA. Part B, Resources 4(2): 2942–2943. 10.1080/23802359.2019.1662744 PMC7707012 33365803

[B46] Wickham H (2016) Data Analysis. In: Wickham H (Ed.) ggplot2: Elegant Graphics for Data Analysis. Springer International Publishing, Cham, 189–201. 10.1007/978-3-319-24277-4_9

[B47] Xie J, Feng J, Guo Y, Ye Y, Li J, Guo B (2019) The complete mitochondrial genome and phylogenetic analysis of *Nerita yoldii* (Gastropoda: Neritidae). Mitochondrial DNA. Part B, Resources 4(1): 1099–1100. 10.1080/23802359.2019.1586485

[B48] Xu Y, Zeng S, Meng Y, Yang D, Yang S (2024) The mitochondrial genome of *Hua aristarchorum* (Heude, 1889)(Gastropoda, Cerithioidea, Semisulcospiridae) and its phylogenetic implications. ZooKeys 1192: 237–255. 10.3897/zookeys.1192.116269 PMC10905624 38433759

[B49] Xu Y-B, Meng Y-Z, Zeng S, Wang H-J, Zhong S, Yang D-Y, Zhou X-P, Glasby CJ (2025) A new species of *Semisulcospira* O. Boettger, 1886 (Gastropoda, Cerithioidea, Semisulcospiridae) from Fujian, China with mitochondrial genome and its phylogenetic implications. Zoosystematics and Evolution 101(1): 17–34. 10.3897/zse.101.136882

[B50] Yang D, Zeng S, Wang Z, Zhang Y, Yang D, Glasby CJ, Hwang JS, Cai L (2024) Molecular systematics of *Perinereis* and an investigation of the status and relationships of the cultured species *Perinereis wilsoni* Glasby & Hsieh, 2006 (Annelida, Nereididae). Zoosystematics and Evolution 100(4): 1297–1314. 10.3897/zse.100.127201

[B51] Zhang D, Gao F, Jakovlić I, Zou H, Zhang J, Li WX, Wang GT (2020) PhyloSuite: An integrated and scalable desktop platform for streamlined molecular sequence data management and evolutionary phylogenetics studies. Molecular Ecology Resources 20(1): 348–355. 10.1111/1755-0998.13096 31599058

