# Supplementary figures and images for: Molecular phylogenetic analysis of Neritona juttingae (Mienis, 1973) (Gastropoda, Cycloneritida, Neritidae) with remarks on the phylogenetic position of the genus Neritona
Source: Zookeys. 2026 Feb 13;1269:129–49. doi: 10.3897/zookeys.1269.164112 (PMC12924057; doi:10.3897/zookeys.1269.164112)

BI tree

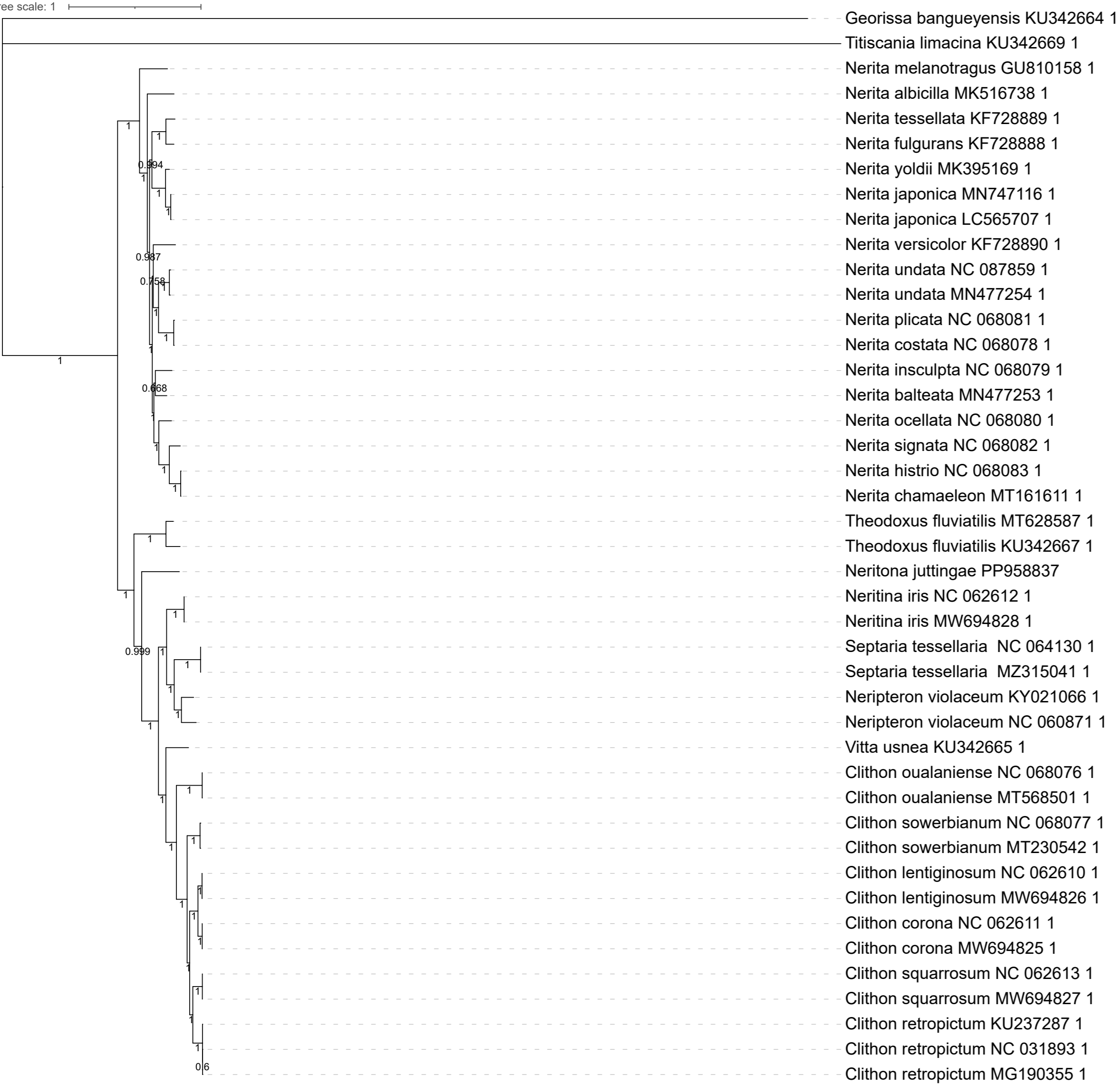

ML tree

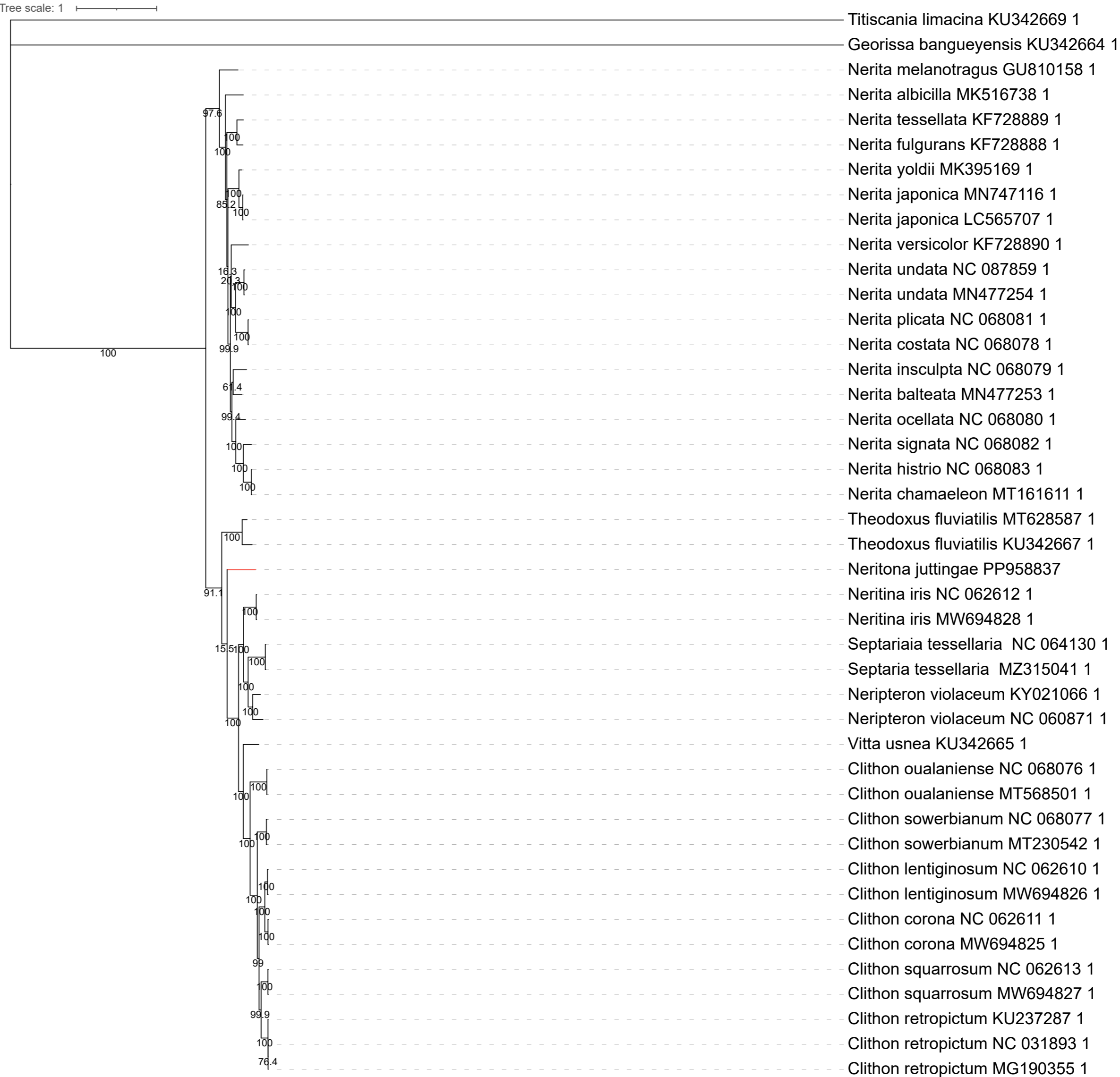

Supplement: Supplementary material 2 — ML and BI from all NCBI mitochondrial genomes (2025-01-10) of Neritidae [file zookeys-1269-129_article-164112__-s002.pdf]

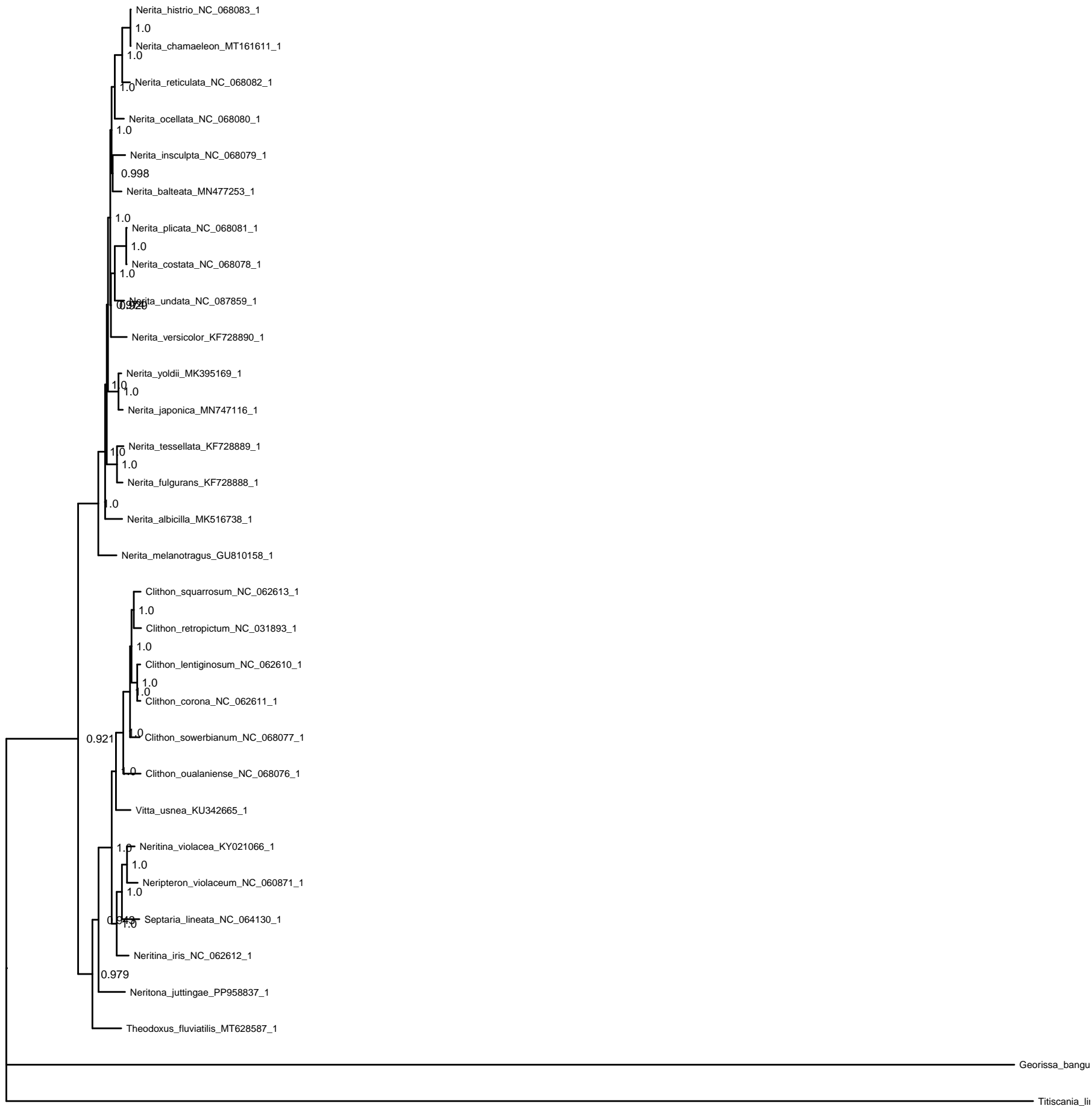

Supplement: Supplementary material 4 — 10 trees from different datasets and tree-building methods [file zookeys-1269-129_article-164112__-s004.zip › Supplementary file 3/13PCGs123_2R_BI_1.tre_tree_with_bootstrap.pdf]

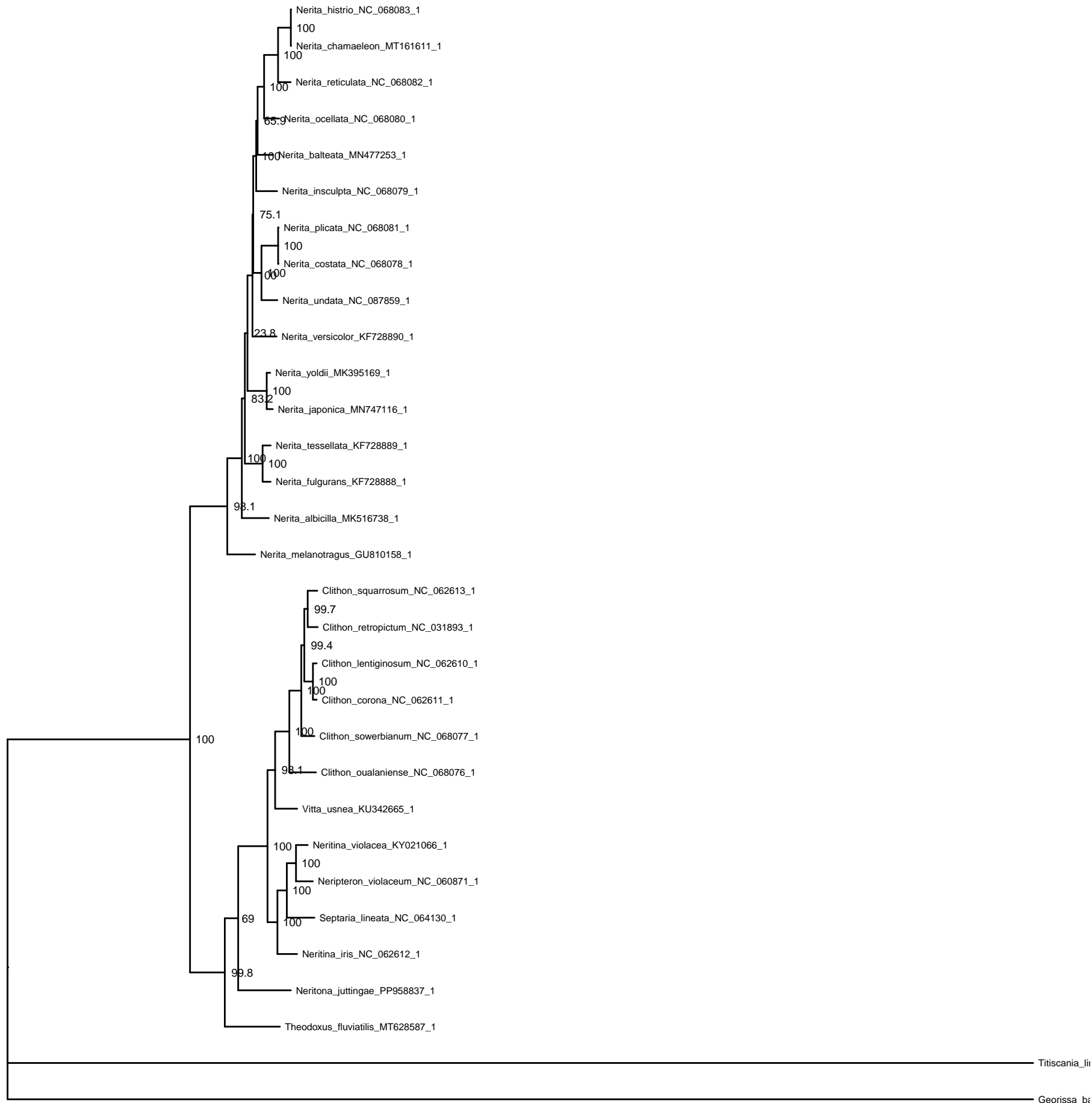

Supplement: Supplementary material 4 — 10 trees from different datasets and tree-building methods [file zookeys-1269-129_article-164112__-s004.zip › Supplementary file 3/13PCGs123_2R_ML_1.treefile_tree_with_bootstrap.pdf]

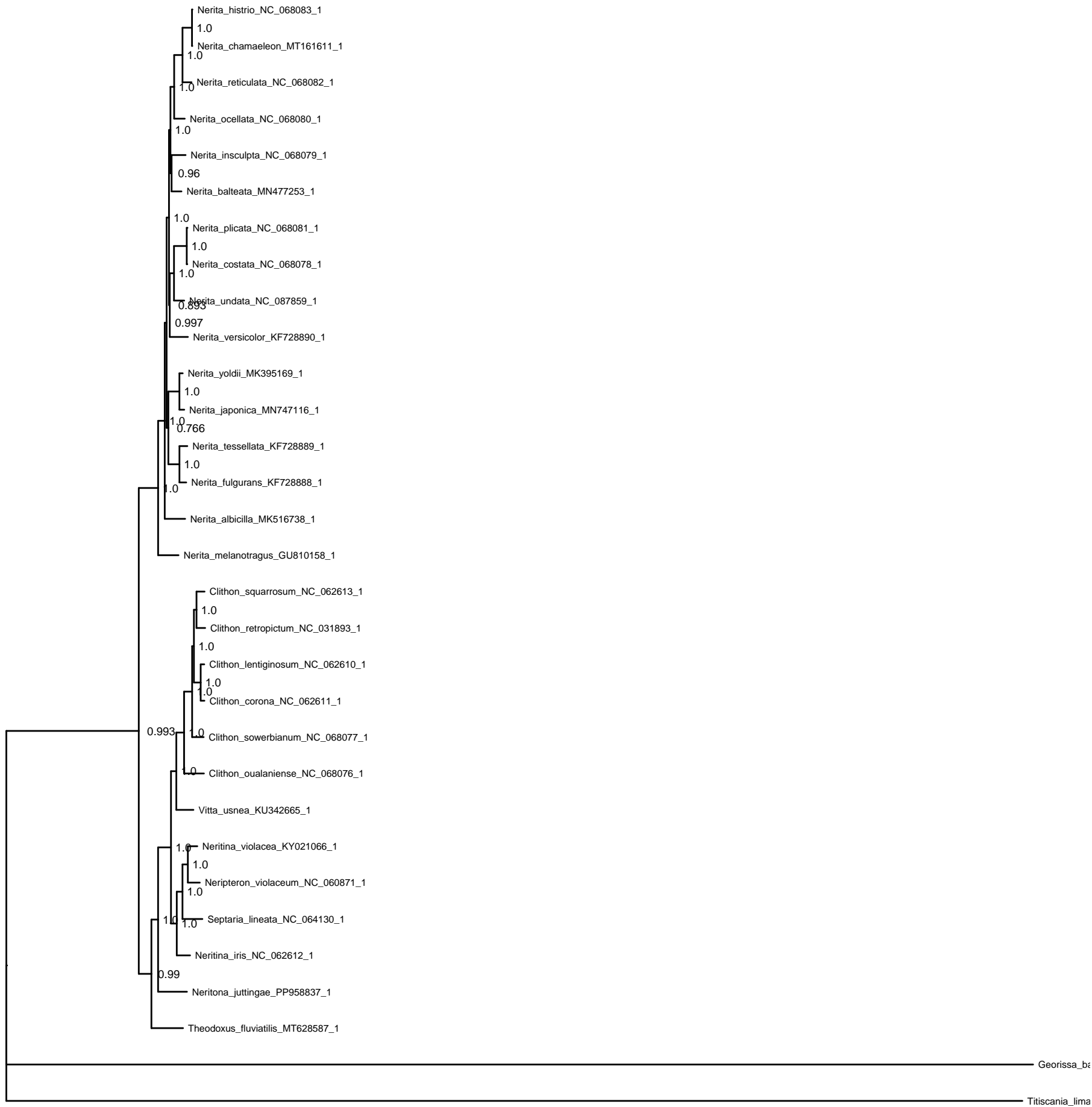

Supplement: Supplementary material 4 — 10 trees from different datasets and tree-building methods [file zookeys-1269-129_article-164112__-s004.zip › Supplementary file 3/13PCGs123_BI_1.tre_tree_with_bootstrap.pdf]

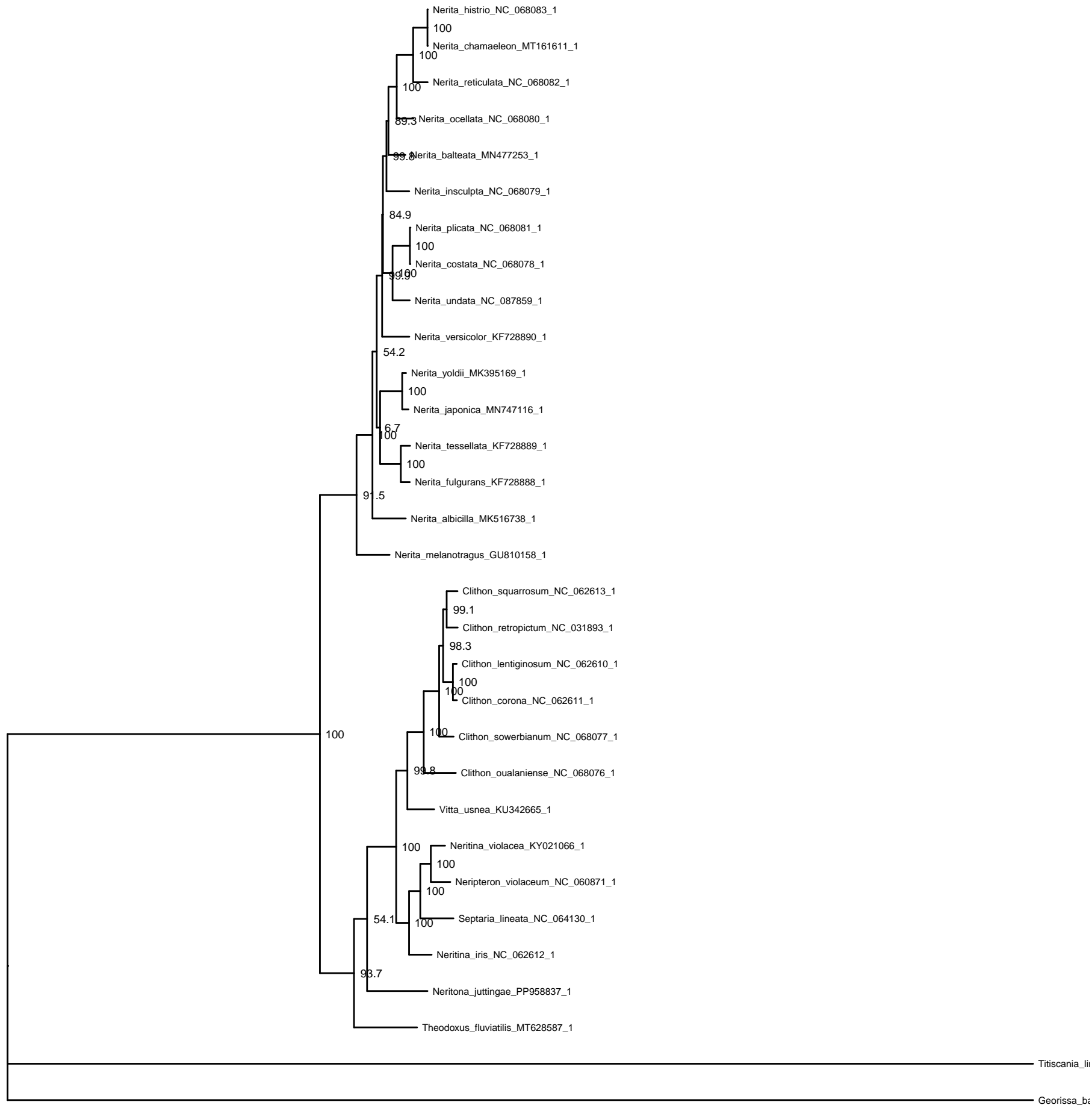

Supplement: Supplementary material 4 — 10 trees from different datasets and tree-building methods [file zookeys-1269-129_article-164112__-s004.zip › Supplementary file 3/13PCGs123_ML_1.treefile_tree_with_bootstrap.pdf]

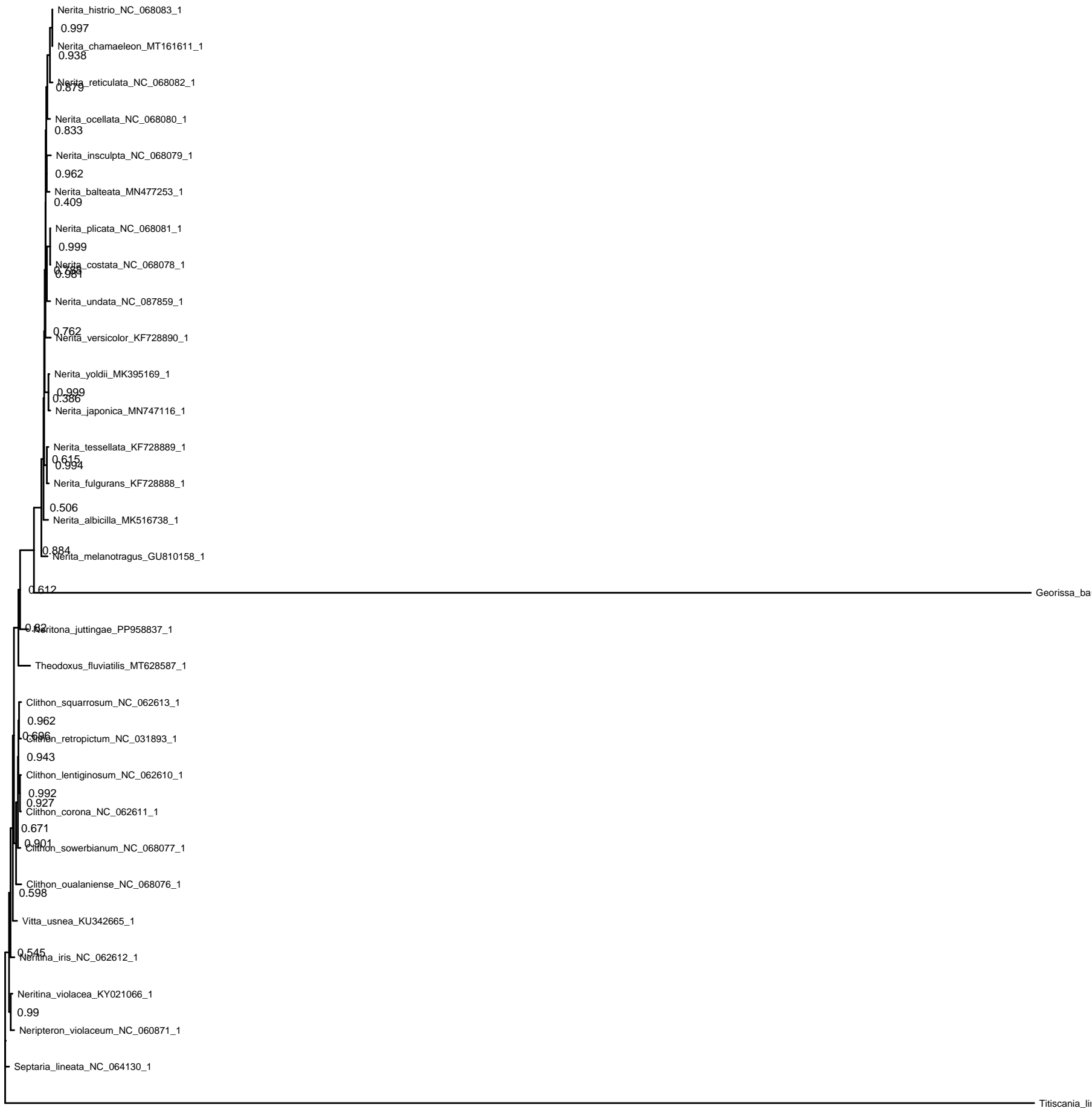

Supplement: Supplementary material 4 — 10 trees from different datasets and tree-building methods [file zookeys-1269-129_article-164112__-s004.zip › Supplementary file 3/13PCGs12_2R_BI_1.tre_tree_with_bootstrap.pdf]

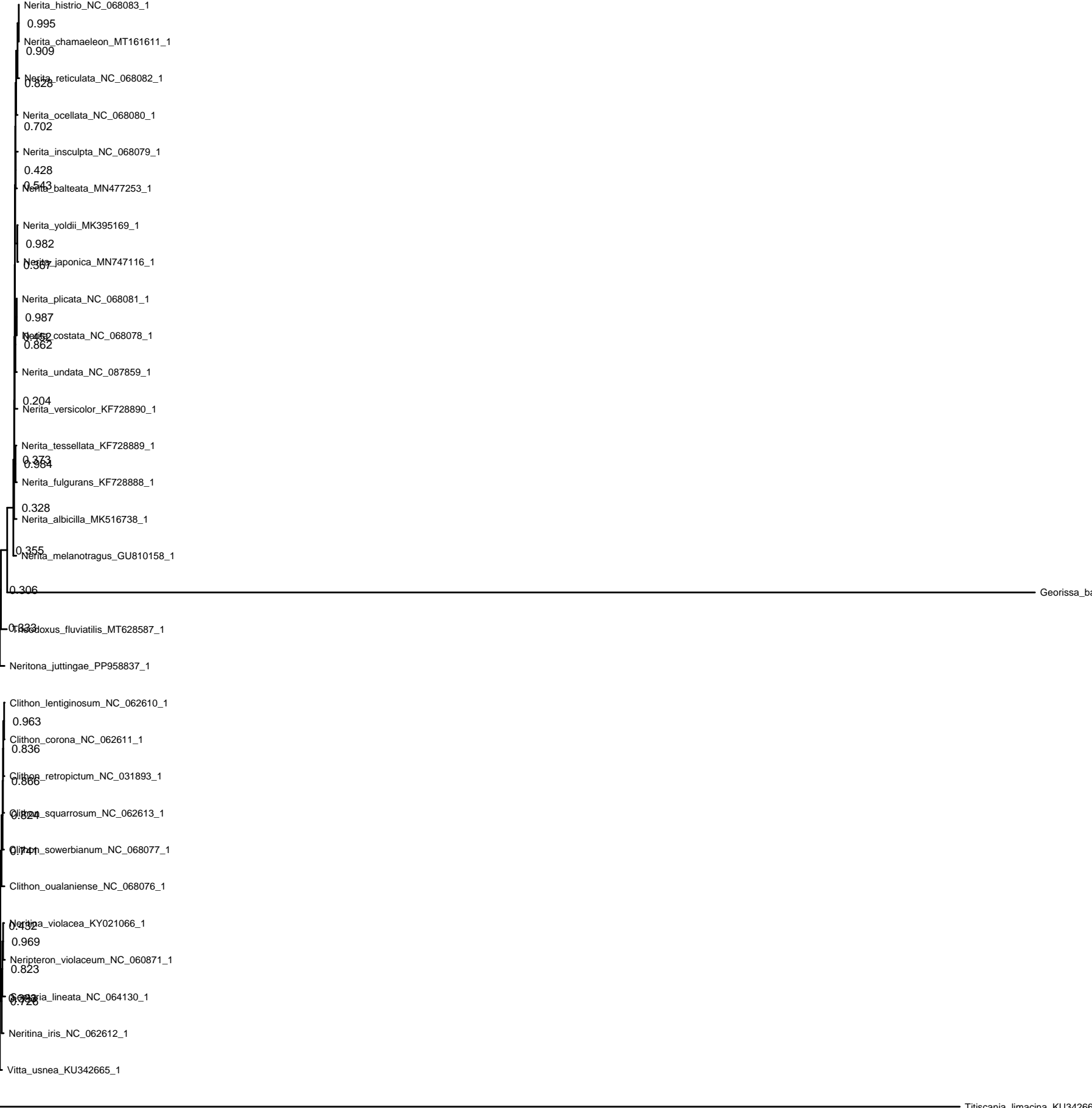

Supplement: Supplementary material 4 — 10 trees from different datasets and tree-building methods [file zookeys-1269-129_article-164112__-s004.zip › Supplementary file 3/13PCGs12_BI_1.tre_tree_with_bootstrap.pdf]

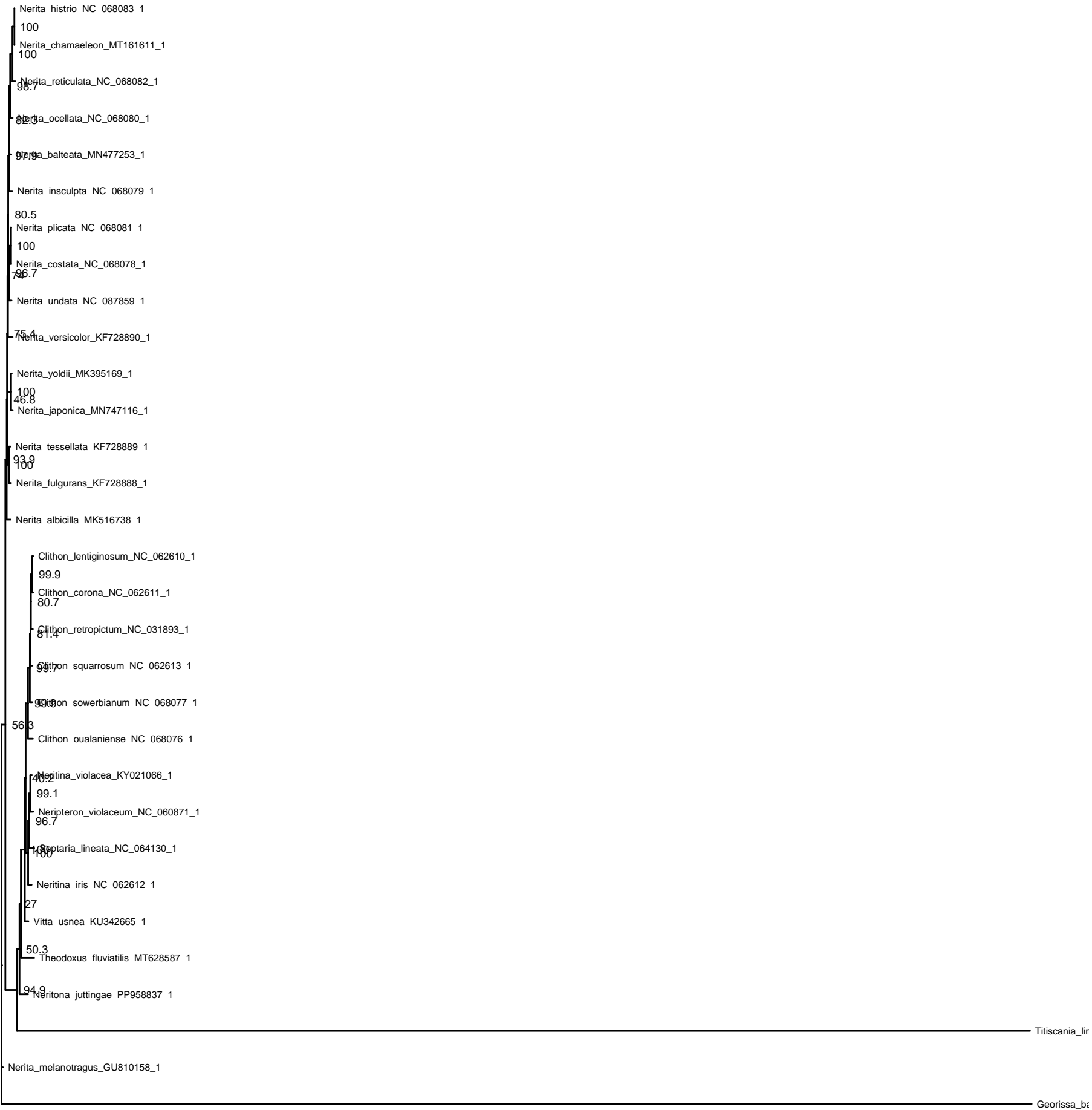

Supplement: Supplementary material 4 — 10 trees from different datasets and tree-building methods [file zookeys-1269-129_article-164112__-s004.zip › Supplementary file 3/13PCGs12_ML_1.treefile_tree_with_bootstrap.pdf]

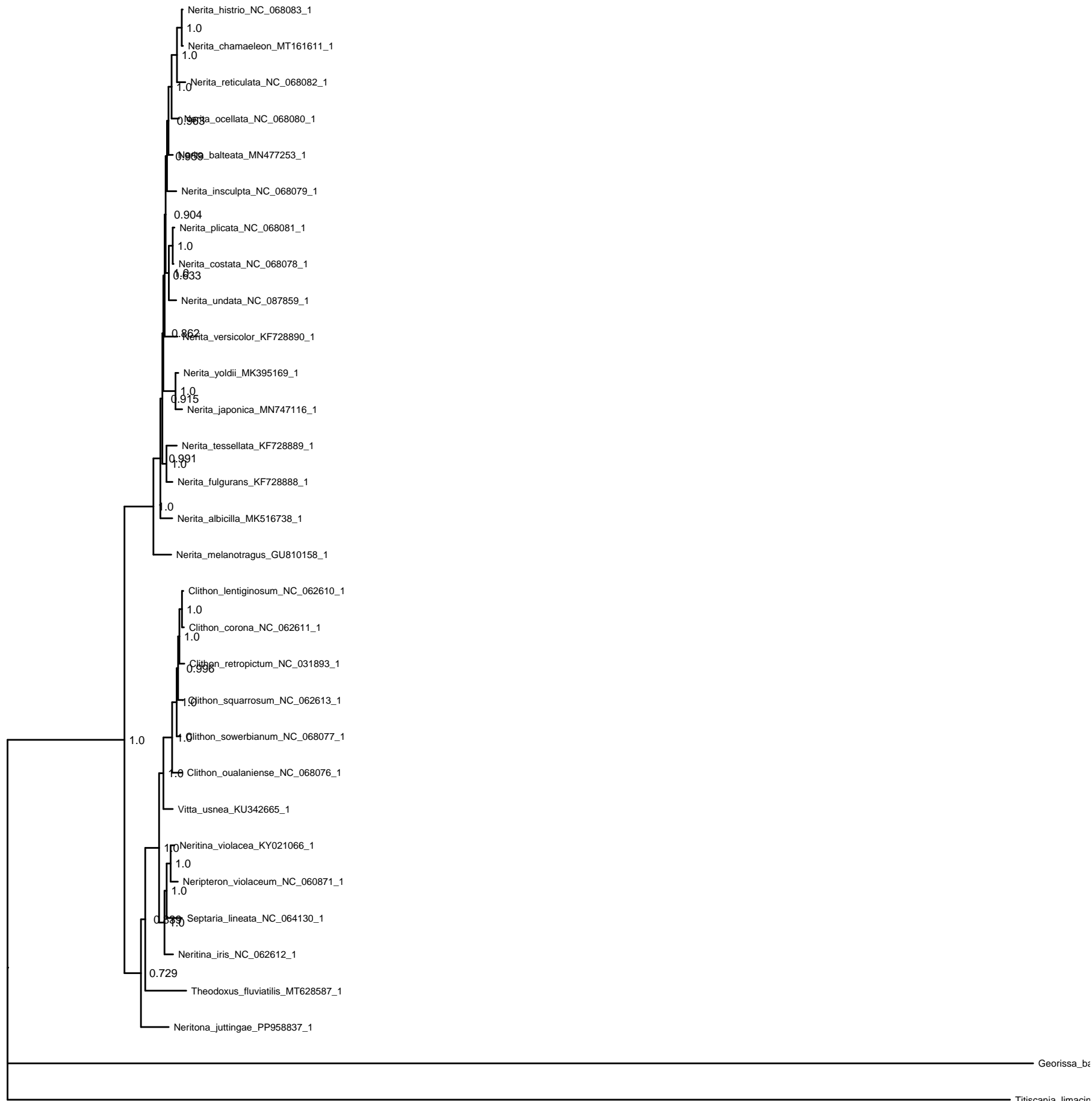

Supplement: Supplementary material 4 — 10 trees from different datasets and tree-building methods [file zookeys-1269-129_article-164112__-s004.zip › Supplementary file 3/13PCGsAA_BI_1.tre_tree_with_bootstrap.pdf]

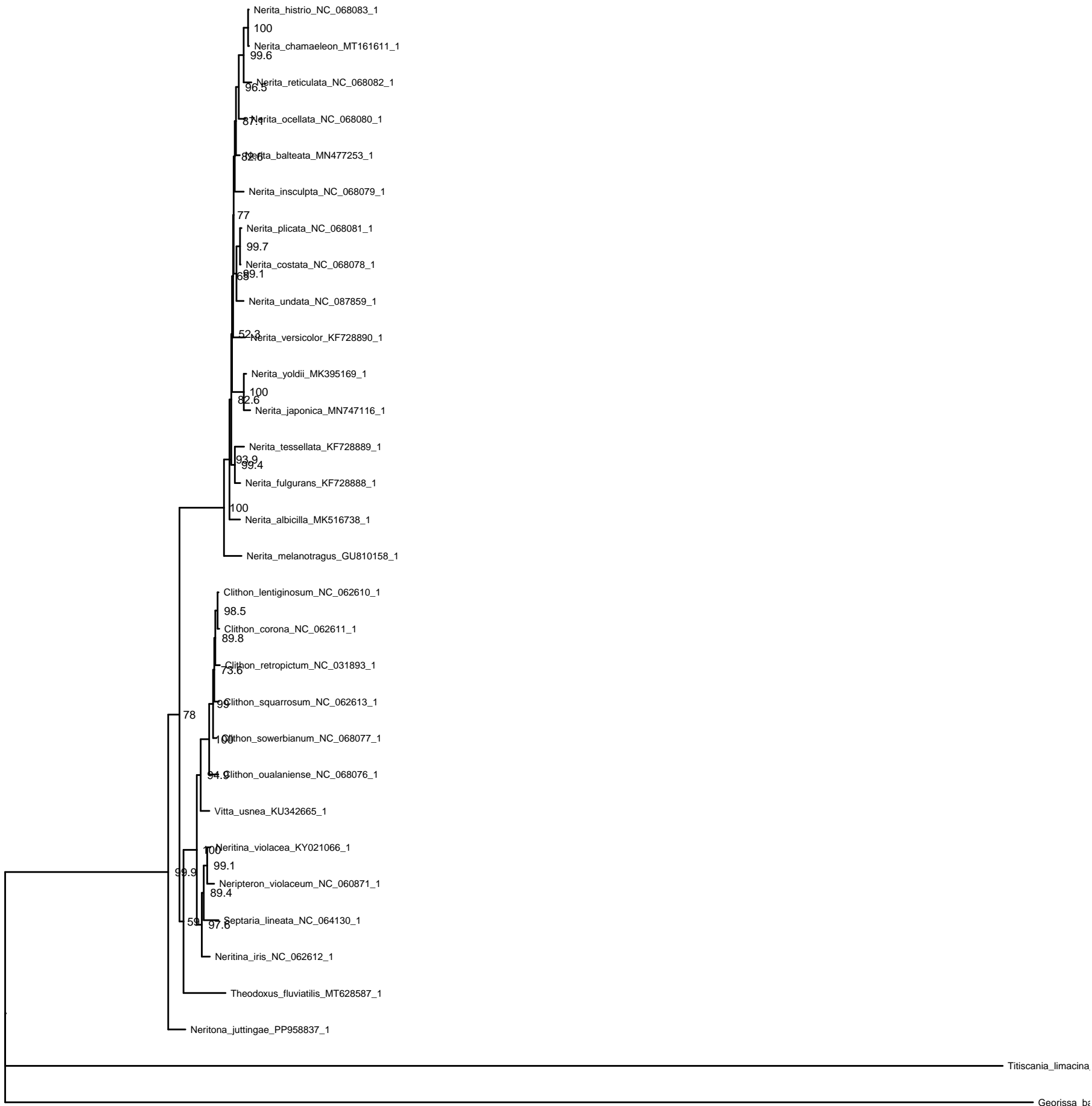

Supplement: Supplementary material 4 — 10 trees from different datasets and tree-building methods [file zookeys-1269-129_article-164112__-s004.zip › Supplementary file 3/13PCgsAA_ML_1.treefile_tree_with_bootstrap.pdf]

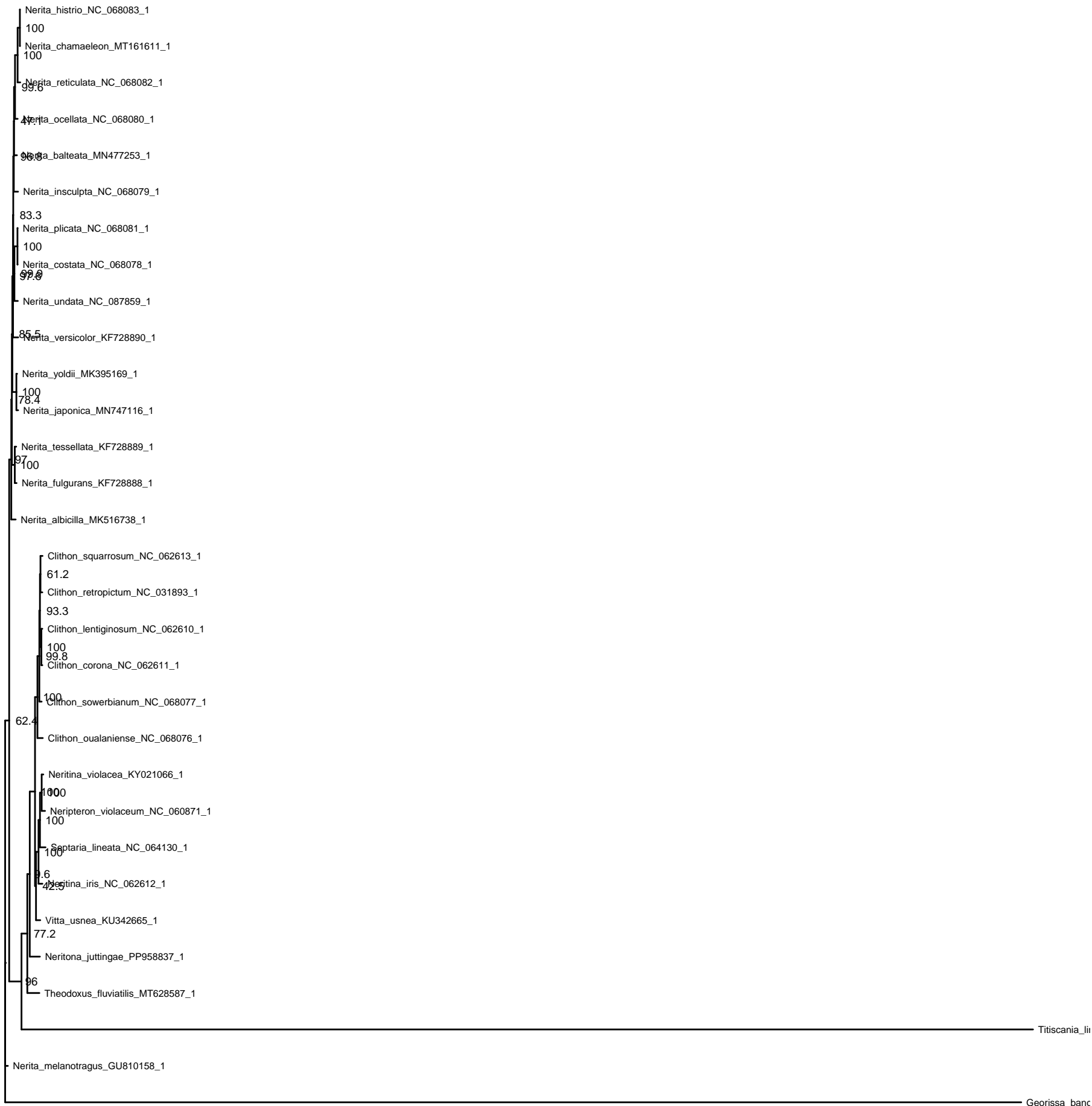

Supplement: Supplementary material 4 — 10 trees from different datasets and tree-building methods [file zookeys-1269-129_article-164112__-s004.zip › Supplementary file 3/13PCGs_2R_ML_1.treefile_tree_with_bootstrap.pdf]

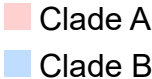

Supplement: Supplementary material 5 — ML tree based on the COI+16S dataset [file zookeys-1269-129_article-164112__-s005.pdf]

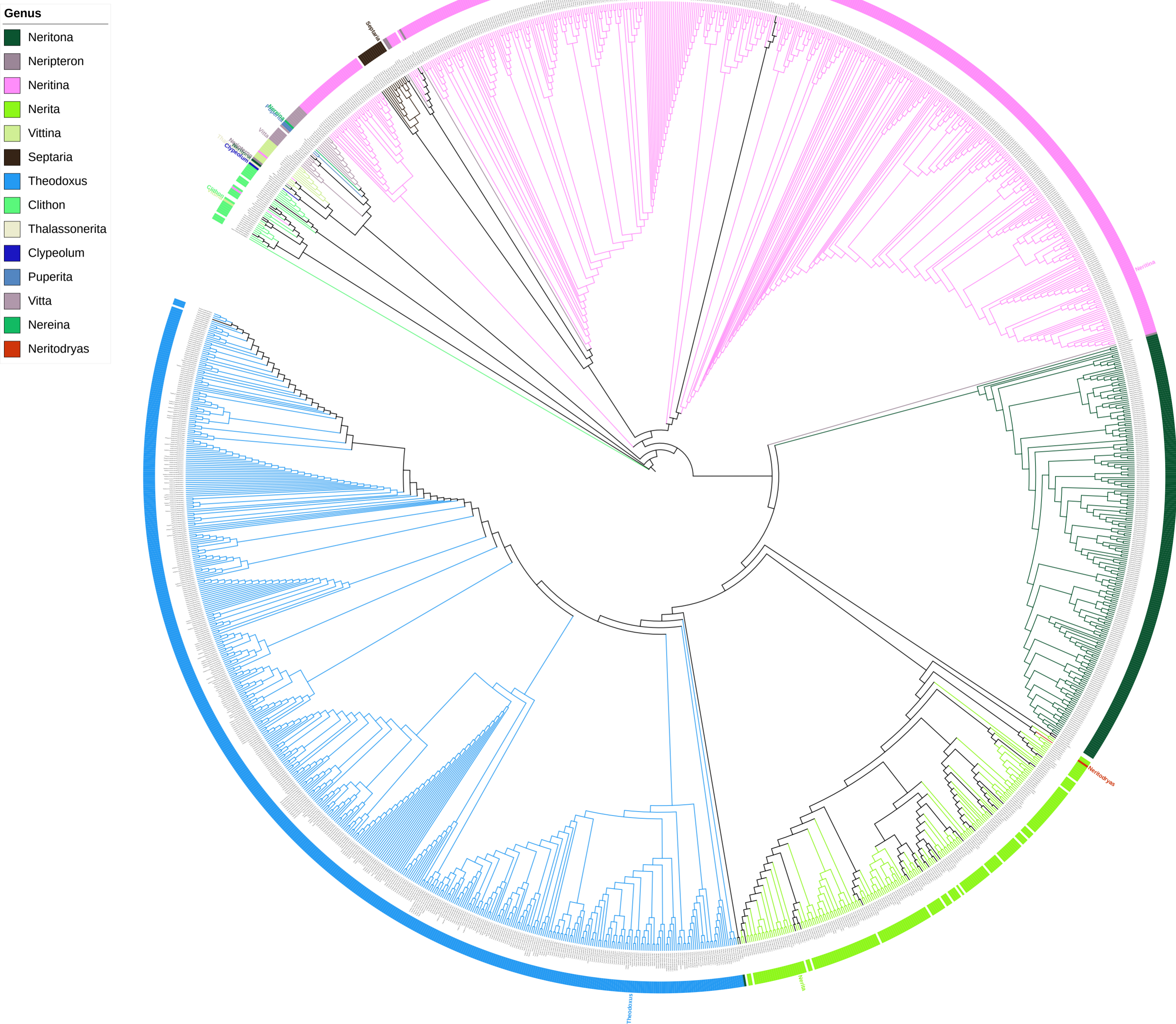

Supplement: Supplementary material 6 — ML tree based on the COI_ex dataset [file zookeys-1269-129_article-164112__-s006.pdf]
