# Supplementary material for: Molecular phylogenetic analysis of Neritona juttingae (Mienis, 1973) (Gastropoda, Cycloneritida, Neritidae) with remarks on the phylogenetic position of the genus Neritona
Source: Zookeys. 2026 Feb 13;1269:129–49. doi: 10.3897/zookeys.1269.164112 (PMC12924057; doi:10.3897/zookeys.1269.164112)
Supplement: Supplementary material 3 — All the mt genes order of Neritidae [file zookeys-1269-129_article-164112__-s003.pdf]

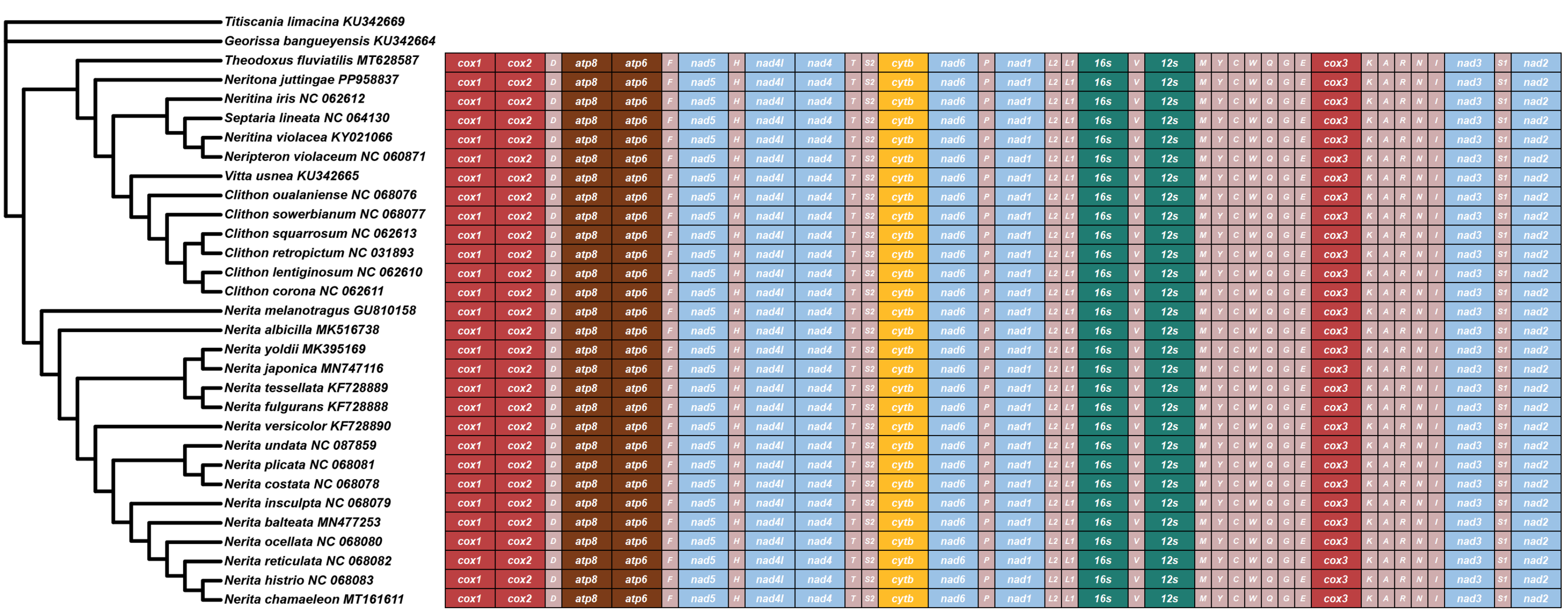
Titiscania limacina KU342669
Georissa bangueyensis KU342664
Theodoxus fluviatilis MT628587
Neritona juttingae PP958837
Neritina iris NC 062612
Septaria lineata NC 064130
Neritina violacea KY021066
Neripteron violaceum NC 060871
Vitta usnea KU342665
Clithon oualaniense NC 068076
Clithon sowerbianum NC 068077
Clithon squarrosum NC 062613
Clithon retropictum NC 031893
Clithon lentiginosum NC 062610
Clithon corona NC 062611
Nerita melanotragus GU810158
Nerita albicilla MK516738
Nerita yoldii MK395169
Nerita japonica MN747116
Nerita tessellata KF728889
Nerita fulgurans KF728888
Nerita versicolor KF728890
Nerita undata NC 087859
Nerita plicata NC 068081
Nerita costata NC 068078
Nerita insculpta NC 068079
Nerita balteata MN477253
Nerita ocellata NC 068080
Nerita reticulata NC 068082
Nerita histrio NC 068083
Nerita chamaeleon MT161611
cox1 cox2 D atp8 atp6 F nad5 H nad4l nad4 T S2 cytb nad6 P nad1 L2 L1 16s V 12s M Y C W Q G E cox3 K A R N I nad3 S1 nad2
